# Disrupting DDB2-DNA Interaction by Lapatinib Enhances Chemotherapy Sensitivity

**DOI:** 10.7150/ijbs.116148

**Published:** 2025-09-23

**Authors:** Shih-Chao Hsu, Yu-Hao He, Yun-Ju Chen, Uyen Nguyen Phuong Le, Pei-Tong Liu, Thanh Kieu Huynh, Yi-Ling Chen, Ya-Ling Wei, Hsin-Chiao Chou, Wei-Chien Huang, Long-Bin Jeng

**Affiliations:** 1 Graduate Institute of Biomedical Sciences, China Medical University, Taichung, 404333, Taiwan.; 2 Department of Surgery, China Medical University Hospital, Taichung, 404327, Taiwan.; 3 Center for Molecular Medicine, China Medical University Hospital, Taichung, 406040, Taiwan.; 4 Cancer Biology and Precision Therapeutics Center, China Medical University, Taichung, 406040, Taiwan.; 5 School of Medicine, I-Shou University, Kaohsiung, 824005, Taiwan.; 6 Department of Medical Research and Department of Pharmacy, E-Da Hospital, Kaohsiung, 824005, Taiwan.; 7 School of Pharmacy, China Medical University, Taichung, 406040, Taiwan.; 8 Department of Medical Research, China Medical University Hsinchu Hospital, Hsinchu, 302056, Taiwan.; 9 Department of Medical Laboratory Science and Biotechnology, Asia University, Taichung, 413305, Taiwan.; 10 School of Medicine, China Medical University, Taichung, 404328, Taiwan.; 11 Organ Transplantation Center, China Medical University Hospital, Taichung, 404327, Taiwan.

**Keywords:** lapatinib, DDB2, chemosensitization, nucleotide excision repair, proteasomal degradation

## Abstract

Chemoresistance remains an obstacle to effective cancer therapy across multiple tumor types. Damaged DNA-binding protein 2 (DDB2), a key component of the nucleotide excision repair (NER) pathway, contributes to chemoresistance by enhancing DNA repair and inhibiting apoptosis. Although the role of DDB2 in tumor progression is context-dependent, its upregulation has been associated with poor prognosis in various malignancies. In this study, elevated DDB2 levels found in breast, liver, cholangiocarcinoma, and lung cancers correlated with reduced patient survival. DDB2 confers resistance to chemotherapeutic agents. Through structure-based virtual screening and molecular dynamics simulations, lapatinib, an FDA-approved EGFR/HER2 inhibitor, was identified as a compound capable of disrupting the DDB2/DNA complex, which was confirmed by the cellular thermal shift assay and chromatin fractionation. Mechanistically, lapatinib binds to the DNA-binding region of DDB2, thereby reducing its chromatin association and promoting proteasomal degradation. Co-treatment with lapatinib and doxorubicin exhibited synergistic cytotoxicity in both cancer cell lines and patient-derived organoids. These findings reveal a previously unrecognized role for lapatinib in targeting DNA repair machinery, supporting its repurposing as a chemosensitizing agent. Our study highlights DDB2 as a critical mediator of chemoresistance and proposes disruption of DDB2-dependent DNA repair as a novel strategy for chemosensitization.

## Introduction

Cancer remains one of the most pressing global challenges of the 21st century, representing a major burden on public health, society, and the economy. It accounts for approximately one in six deaths (16.8%) and nearly one in four deaths from non-communicable diseases (22.8%) worldwide 1. Despite significant progress in targeted therapies and immunotherapy, chemotherapy continues to serve as a cornerstone of first-line treatment for many malignancies, including lung, breast, and colorectal cancers. However, therapeutic efficacy is often limited by tumor heterogeneity and the eventual development of drug resistance. Furthermore, prolonged chemotherapy can lead to severe systemic toxicity, resulting in multi-organ dysfunction and substantial physical and psychological stress for patients 2.

A critical determinant of chemotherapeutic response is the DNA damage response (DDR), which governs cellular fates such as apoptosis, cell cycle arrest, senescence, and DNA repair following genotoxic stress 3. Higher expression of DDR genes correlates with stemness and poor survival rate in triple-negative breast cancer (TNBC) patients 4. Chemoresistance frequently arises from the efficient activation of DNA repair pathways that enable tumor cells to survive despite sustained DNA damage 5. Among these, nucleotide excision repair (NER) plays a pivotal role in repairing bulky DNA lesions induced by agents such as UV light, cisplatin, and doxorubicin 6, 7. Compared to other DNA repair mechanisms, such as base excision repair (BER), which handles small oxidative lesions, or homologous recombination (HR), which repairs double-strand breaks, NER addresses a broader range of helix-distorting damage that is directly relevant to the action of many front-line chemotherapeutics 8.

Within the NER machinery, Damaged DNA-binding protein 2 (DDB2) functions as an essential initiator of NER by recognizing DNA lesions and facilitating the recruitment of the downstream repair complex, including xeroderma pigmentosum group C protein (XPC) and other NER regulators 9. Mutations in DDB2 can disrupt DDB2-DNA or DDB2-DDB1 interactions, leading to impaired NER activity 10. Elevated expression of DDB2 has been linked to enhanced DNA repair capacity and poor clinical responses to DNA alkylating or interstrand cross-link agents in melanoma 11, glioblastoma 12, lung 13, and breast cancers 14. Notably, DDB2 overexpression promotes proliferation and colony formation in ERα-positive breast cancer cells 15, and protects triple-negative breast cancer (TNBC) cells from PARP inhibitor (PARPi) therapy by enhancing HR-mediated repair of double-strand breaks 16. Furthermore, mutant p53 has been shown to confer chemoresistance by altering its transcriptional preference from *lincRNA-p21* to *DDB2*, depending on ER status 17, 18. Moreover, a preclinical study also indicated the essential role of DDB2 in conferring pancreatic cancer cells resistant to radiotherapy 19. These findings suggest that selectively inhibiting NER by targeting DDB2 is a potential strategy to sensitize tumors to DNA-damaging agents, and could exploit a tumor-specific vulnerability not shared by normal cells.

However, the function of DDB2 appears to be context-dependent across different cancers. Beyond its role in the repair of damaged DNA, DDB2 has been implicated in repressing the subpopulation of cancer stem cells by acting as a transcriptional regulator 4, 20, 21. For instance, DDB2 knockdown promotes proliferation in gastric cancer cells 22, while in ovarian cancer, DDB2 sensitizes cells to cisplatin-induced apoptosis by downregulating Bcl-2 9. In colorectal and pancreatic cancers, DDB2 inhibits epithelial-to-mesenchymal transition (EMT), suggesting a tumor-suppressive role in metastasis 19, 23. These diverse and sometimes contradictory roles underscore the complexity of DDB2's function in cancer biology.

Given these observations, we investigated the role of DDB2 in mediating chemoresistance across multiple cancer types and employed *in silico* screening to identify potential inhibitors that disrupt its DNA-binding activity. Our study identifies DDB2 as a critical regulator of DNA repair-driven chemoresistance in breast, liver, cholangiocarcinoma, and lung cancers. Notably, we found that lapatinib, a clinically approved dual EGFR/HER2 inhibitor for HER2-positive metastatic breast cancer 24, effectively impairs the DNA-binding activity of DDB2 and enhances chemosensitivity by suppressing DNA repair mechanisms. These findings provide compelling evidence that repurposing lapatinib to target DDB2-DNA interaction could potentiate chemotherapy responses across diverse malignancies.

## Materials and Methods

### Cell culture

Breast cancer cell lines MCF7 (RRID: CVCL_0031), T-47D (RRID: CVCL_0553), BT-474 (RRID: CVCL_0179), SK-BR-3 (RRID: CVCL_0033), MDA-MB-468 (RRID: CVCL_0419), MDA-MB-231 (RRID: CVCL_0062), BT549 (RRID: CVCL_1092), Hs 578T (RRID: CVCL_0332), HBL-100 (RRID: CVCL_4362), and MDA-MB-157 (RRID: CVCL_0618), and the liver cancer cell lines HepG2 (RRID: CVCL_0027), Hep3B (RRID: CVCL_0326), and the cholangiocarcinoma cell line KKU-100 (RRID: CVCL_3996) were cultured in Dulbecco's Modified Eagle Medium: Nutrient Mixture F-12 (DMEM/F12, HyClone™, Thermo Fisher Scientific Inc., Waltham, MA, USA) supplemented with 10% fetal bovine serum (FBS, Gibco, Thermo Fisher Scientific Inc., Waltham, MA, USA) and HyClone™ Penicillin-Streptomycin Solution. The normal breast cell line MCF-10A (RRID: CVCL_0598) was cultured in DMEM/F12 supplemented with 5% horse serum (Gibco), HyClone™ Penicillin-Streptomycin Solution and other supplements including 1.05 mM calcium chloride anhydrous (Sigma-Aldrich, Merck KGaA, Darmstadt, Germany), 0.1 μg/mL cholera toxin, 10 μg/mL insulin (Sigma-Aldrich), 20 ng/mL human epidermal growth factor (EGF) (Sigma-Aldrich), and 0.5 μg/mL hydrocortisone (Sigma-Aldrich). The medium for some liver cancer cell lines, such as TONG (RRID: CVCL_V640), PLC/PRF/5 (RRID: CVCL_0485), and Mahlavu (RRID: CVCL_0405), was cultured in Dulbecco's Modified Eagle Medium (DMEM, HyClone™, Thermo Fisher Scientific Inc, Waltham, MA, USA) supplemented with 10% FBS and HyClone™ Penicillin-Streptomycin Solution, and the Huh7 liver cancer cell line (RRID: CVCL_0336) was cultured in DMEM supplemented with 1% L-glutamine. Two cholangiocarcinoma cell lines, HuCCT1 and SNU-1196 were cultured in Roswell Park Memorial Institute (RPMI) 1640 (HyClone™, Thermo Fisher Scientific Inc., Waltham, MA, USA) supplemented with 10% FBS (Gibco, Thermo Fisher Scientific Inc., Waltham, MA, USA) and penicillin-streptomycin solution (HyClone™). All cell lines were purchased from the American Type Culture Collection (ATCC) and incubated at 37°C in a humidified incubator containing 5% CO_2_ and tested for mycoplasma contamination using the MycoAlert™ Mycoplasma Detection Kit (LT07-318, Thermo Fisher Scientific Inc., Waltham, MA, USA).

### Inhibitors and reagents

Carboplatin (41575-94-4) and (S)-MG132 (133407-82-6) were purchased from Cayman Chemical (Michigan, USA). Cisplatin (cis-diammineplatinum (II), P4394) and doxorubicin hydrochloride (Sigma-Aldrich, D1515) were purchased from Merck KGaA (Darmstadt, Germany). Clarity™, Amersh™, or Millipore Enhanced chemiluminescence (ECL) was purchased from Bio-Rad Laboratories, Inc., (Hercules, CA, USA), GE Healthcare Life Science (Pittsburgh, PA, USA), or Merck KGaA (Darmstadt, Germany), respectively. Lipo-doxorubicin was kindly provided by a clinical doctor.

### Cell viability assay

Cells (3×10^3^) cultured in 96-well plates were treated with different drug concentrations for 48 hours. The culture medium was then replaced with serum-free medium containing 5X Sigma-Aldrich MTT solution. After incubation for 3 hours, the cells were lysed with DMSO and the optical density (OD) at 550 nm was measured using an ELISA reader. The IC_50_ values of doxorubicin and lapatinib were determined by the MTT assay. The combination index (CI) was calculated using the formula: CI = (concentration of drug A/IC_50_ of drug A alone) + (concentration of drug B/IC_50_ of drug B alone).

### Western blot analysis

Total protein lysate concentration was determined by the Bradford protein assay (Bio-Rad Laboratories, Inc., Hercules, CA, USA), in which 30 μg of protein lysate was heated at 95°C for 5 minutes in sample buffer. Denatured proteins were separated by SDS-PAGE with running buffer and transferred to PVDF membranes (0.45 μM, Millipore, Merck KGaA, Darmstadt, Germany) or NC membranes (0.22 μM, Amersh™, GE Healthcare Life Science, Pittsburgh, PA, USA) with transfer buffer. The transferred membrane was blocked with 5% milk or BSA in TBST buffer and stained with the indicated primary antibodies overnight at 4°C, followed by incubation with HRP-conjugated secondary antibodies. ECL signals were detected using a ChemiDoc™ Touch Imaging System (Bio-Rad).

### Antibodies

Antibodies against DDB2 (#5416, RRID: AB_10694497), Histone H3 (#9715, RRID: AB_331563), p-HER2 (Y1221/1222, #2243, RRID: AB_490899), p-EGFR (Y1068, #2236, RRID: AB_331792), Ac-p53 (K382, #2525S, RRID: AB_330083), and PARP (#9542, RRID: AB_2160739), were purchased from Cell Signaling Technology, Inc., (Beverly, MA, USA). Antibodies to α-tubulin (T5168, RRID: AB_477579), β-actin (A2228, RRID: AB_476697), and p21^WAF1^ (Calbiochem, OP64, RRID: AB_2335868) were purchased from Merck KGaA (Darmstadt, Germany). Antibody against Caspase3 (Imgenex IMG-144A, RRID: AB_316677) was purchased from Novus Biologicals, LLC., (Centennial, CO, USA). Antibody against p-Histone H2AX (Ser139, AF2288, RRID: AB_2114989) was purchased from R&D Systems Inc., (Minneapolis, MN, USA). Antibodies to DDB1 (sc-25367, RRID: AB_639050), HA-probe (sc-7392, RRID: AB_627809), HER2 (Neu, sc-393712, RRID: AB_2810840), EGFR (sc-03, RRID: AB_631420), p53 (sc-126, RRID: AB_628082), and XPC (sc-74410, RRID: AB_1131407) were purchased from Santa Cruz Biotechnology, Inc., (CA, USA).

### Triton extraction assay

Following treatment with chemo-drugs, cells were lysed using Triton extraction buffer (100 mM NaCl, 300 mM sucrose, 3 mM MgCl_2_, 10 mM PIPES pH 6.8, 1 mM EGTA pH 6.8, 0.2% Triton X-100). The lysate, supplemented with 1 mM NaVO_4_, 1 mM PMSF, 10 mM NaF, and 1 ng/mL aprotinin, was kept at 4°C for 30 minutes. The Triton-extractable fraction (chromatin-free protein) was separated as the supernatant, while the Triton-resistant fraction (chromatin-bound protein) was collected as the pellet. The latter was washed twice with Triton extraction buffer before further lysis with NETN buffer (20 mM Tris-HCl pH 8.0, 150 mM NaCl, 1 mM EDTA, 0.5% NP-40) containing the same additional components. Both fractions were used to detect the free and DNA-bound forms of DDB2.

### Comet assay

A basal layer of 1% NMA gel was overlaid with a second layer of 1.5% LMPA gel mixed with chemotherapy-treated cells (1×10^5^) at a 2:1 ratio. This was covered with 1.5% LMPA gel mixed with PBS. After solidification on ice, the slides were treated with 50 μM H_2_O_2_/PBS and lysis solution (2.5 M NaCl, 200 mM NaOH, 100 mM EDTA, 10 mM Tris-HCl pH 10.0, 1% Triton X-100, 10% DMSO) overnight at 4°C. Electrophoresis unwound the DNA, and the resulting cells were stained with ethidium bromide (EtBr). Fluorescence microscopy was used to visualize damaged cells, and Comet Assay III software was used to calculate the tail moment.

### Flow cytometry analysis

Apoptotic cell stages were identified using the Annexin V-FITC Apoptosis Kit. Annexin V and propidium iodide stain early and late apoptotic cells and necrotic cells, respectively. Analysis was performed on the FACSVerse™ flow cytometer.

### Clonogenic assay

As described previously 25, cells (3×10^3^) were seeded in 6-well plates and treated with different concentrations. After 14 days, colonies were stained with crystal violet and quantified using ImageJ software.

### Cellular Thermal Shift Assay (CETSA)

Cell lysate or recombinant DDB2 protein is treated with or without lapatinib at increasing temperatures (40°C to 70°C) followed by western blot analysis to assess the stability of DDB2.

### Molecular Docking and Dynamics Simulations

Using the DDB2-DNA (PDB: 4E54) schematic from the Protein Data Bank, more than a thousand FDA-approved drugs were docked to the DNA-binding region of DDB2 using iGEMDOCK v2.1 26, 27 and the interaction was analyzed by SiMMap analysis 28 and BIOVIA Discovery Studio software (DS2022) (https://www.3ds.com/products/biovia/discovery-studio) (RRID: SCR_015651). Several steps were completed for processing the molecular dynamics (MD) simulations, including solvation, standard dynamics cascade, dynamics production, and trajectory analysis. The MD simulation processes were repeated four times to obtain the average values. In the solvation stage, the minimum distance from the molecular boundary was 15 Å, and the total simulation time for production was 100 nanoseconds (ns) with an interval of 10 picoseconds (ps). The Root Mean Square Deviation (RMSD) and Root Mean Square Fluctuation (RMSF) results were performed to illustrate the stability of the interaction. The docking results are visualized in a 3D structure by using PyMOL software (RRID: SCR_000305).

### Clinical specimen for organoid

Breast cancer tissue samples for the establishment of organoid-like cells were obtained from Chung Shan Medical University Hospital, Taichung, Taiwan. The sample collection included unselected subtypes, and the samples were used according to a protocol approved by the Institutional Review Board of Chung Shan Medical University Hospital, Taichung, Taiwan (CS2-18150). Tumor organoids derived from 4 patient tumor samples (BRCA) were cultured as described 29. Organoids were maintained under standardized culture conditions, and only passages within a defined range (e.g., P2-P5) were used to ensure consistency. Organoid viability was analyzed using the CellTiter-Glo 3D Cell Viability Assay (Promega Corporation, Madison, WI, USA) (RRID: SCR_006724) in biological triplicate.

### Statistical analysis

Student's *t*-test was used to analyze differences between two categorical variables. Results are presented as mean ± SD (n≥3), and a p-value < 0.05 is considered statistically significant. Statistical analyses were performed with GraphPad Prism 9.

## Results

### DDB2 is a crucial determinant of poor outcomes across diverse cancer types

**Figure 1:**
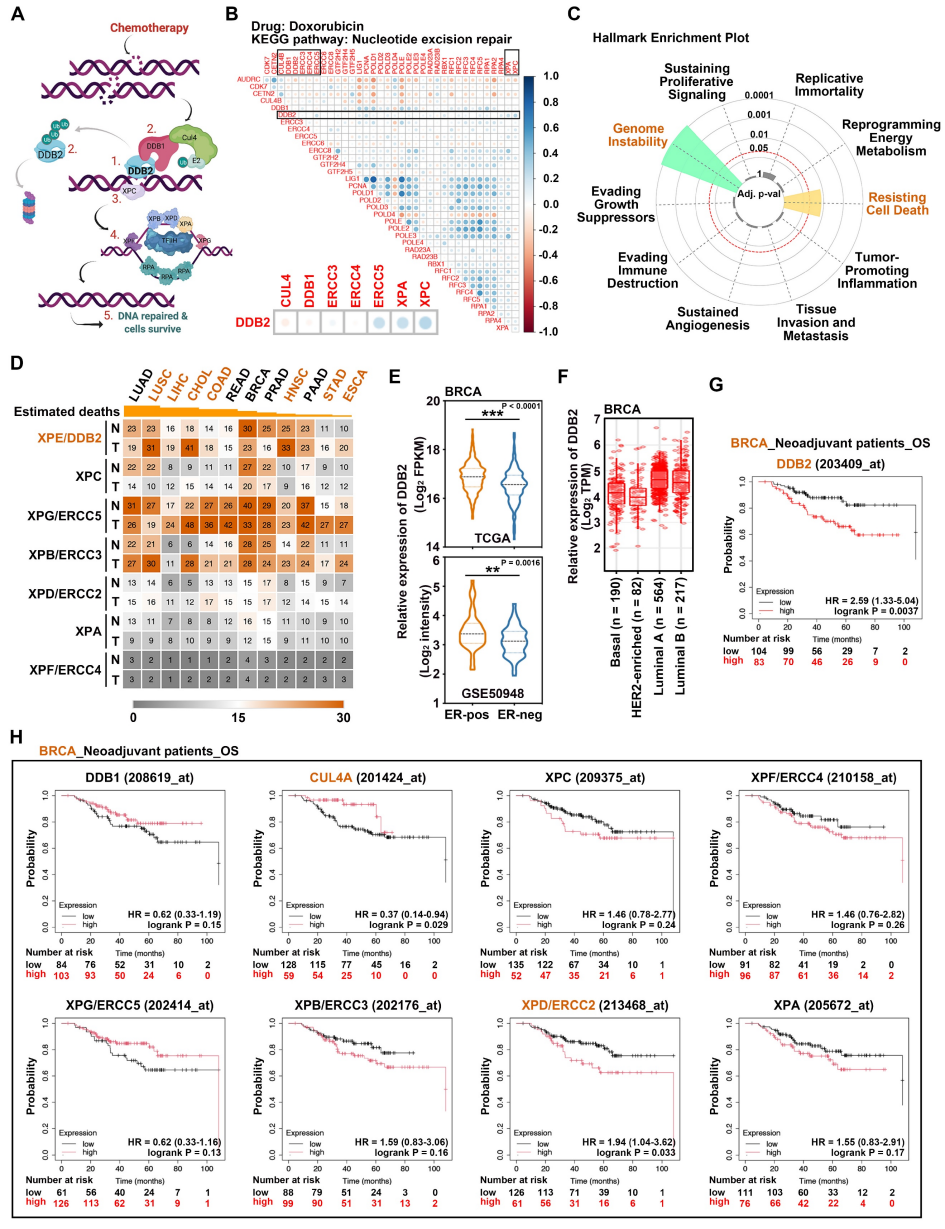
** Pan-cancer analysis of DDB2 expression.** (A) Illustration of the steps of NER. (B) The correlation matrix chart was analyzed by using ROC Plotter (https://www.rocplot.org). (C) Hallmark Enrichment Plot (https://cancerhallmarks.com) was used to identify the functions of NER-related proteins. (D) Gene expression was analyzed by GEPIA (http://gepia.cancer-pku.cn, RRID:SCR_018294) and the heatmap was created by Morpheus-Broad Institute (https://software.broadinstitute.org/morpheus/, RRID:SCR_017386). The cancer type sequence was arranged by the ranking of estimated deaths in Taiwan (https://www.mohw.gov.tw/). The abbreviation of cancer types: LUAD (lung adenocarcinoma), LUSC (lung squamous cell carcinoma), LIHC (liver hepatocellular carcinoma), CHOL (cholangiocarcinoma), COAD (colon adenocarcinoma), READ (rectum adenocarcinoma), BRCA (breast cancer), PRAD (prostate adenocarcinoma), HNSC (head and neck squamous cell carcinoma), PAAD (pancreatic adenocarcinoma), STAD (stomach adenocarcinoma), and ESCA (esophageal carcinoma). (E) The DDB2 expression was analyzed in estrogen receptor-positive and negative breast tumors from TCGA cohort (ER^+^ n=1818, ER^-^ n=511, http://cancergenome.nih.gov/, RRID: SCR_003193) and GSE50948 datasets (ER^+^ n=52, ER^-^ n=104). (F) DDB2 expression in different BRCA subtypes was analyzed by TIMER (https://cistrome.shinyapps.io/timer/). (G-H) Kaplan-Meier plotter (https://kmplot.com/analysis/, RRID: SCR_018753) was used to analyze NER gene survival in neoadjuvant BRCA patients.

DDB2 plays a central role in initiating NER by facilitating the sequential recruitment of DNA repair proteins in response to genotoxic stress (Figure 1A) 4. Its induction by chemotherapeutic agents, such as doxorubicin, has been implicated in the development of chemoresistance through enhanced DNA repair mechanisms 17. To further explore this association, an *in-silico* analysis using the datasets of solid tumors from the Kyoto Encyclopedia of Genes and Genomes (KEGG) revealed a consistent co-upregulation of DDB2 and key NER pathway genes upon treatment with doxorubicin (Figure 1B), cisplatin, or 5-fluorouracil (5-FU) (Supplementary Figure S1A and S1B). This correlation was evident in the ROC Plotter correlation matrix 30, suggesting a shared response pattern to chemotherapeutic stress. Additionally, NER gene upregulation was strongly associated with markers of genomic instability and resistance to cell death in Hallmark Enrichment Plot analysis (Figure 1C) 31.

To assess the oncogenic relevance of DDB2, we analyzed its basal expression across normal and tumor tissues. Among the top ten cancer types contributing to cancer-related mortality, DDB2 was significantly overexpressed in tumor tissues compared to matched normal tissues in lung squamous cell carcinoma (LUSC), liver hepatocellular carcinoma (LIHC), cholangiocarcinoma (CHOL), colon adenocarcinoma (COAD), head and neck squamous cell carcinoma (HNSC), stomach adenocarcinoma (STAD), and esophageal carcinoma (ESCA), as revealed by Gene Expression Profiling Interactive Analysis (GEPIA) 32 (Figure 1D and Supplementary Figure S2A) and in TNMplot 33 (Supplementary Figure S2B). Interestingly, other core NER regulators did not show significant differential expression in these cancer types, with the exception of CHOL and LIHC (Supplementary Figure S3). In breast cancer (BRCA), DDB2 mRNA was enriched in ER-positive tumors within The Cancer Genome Atlas (TCGA) and GSE50948 datasets (Figure 1E), particularly in luminal subtypes (Figure 1F). However, DDB2 expression was not significantly elevated in overall BRCA tumor tissues compared to normal, suggesting that its contribution to chemoresistance may arise from chemotherapy-induced upregulation rather than constitutive overexpression 17.

Prompted by the broad expression of DDB2 across malignancies, we further examined its association with clinical outcomes and chemotherapeutic response. High DDB2 expression correlated with significantly reduced overall survival (OS) in BRCA patients who received neoadjuvant chemotherapy (Figure 1G), as did other NER regulators, with the exception of CUL4A (Figure 1H), based on Kaplan-Meier plotter analysis 34. A similar inverse relationship between DDB2 expression and OS was observed in LUAD (Supplementary Figure S4A), LUSC (Supplementary Figure S4B), and COAD (Supplementary Figure S4C) patients with neoadjuvant chemotherapy, as well as in virus-associated LIHC (Supplementary Figure S4D). Moreover, elevated DDB2 expression was marginally associated with poorer disease-free survival (DFS) in CHOL (Supplementary Figure S4E) and HNSC (Supplementary Figure S4F). Together, these findings highlight DDB2 as a key modulator of chemotherapy response and a potential driver of disease progression across multiple cancer types.

### DDB2 confers chemoresistance through enhanced DNA repair

**Figure 2:**
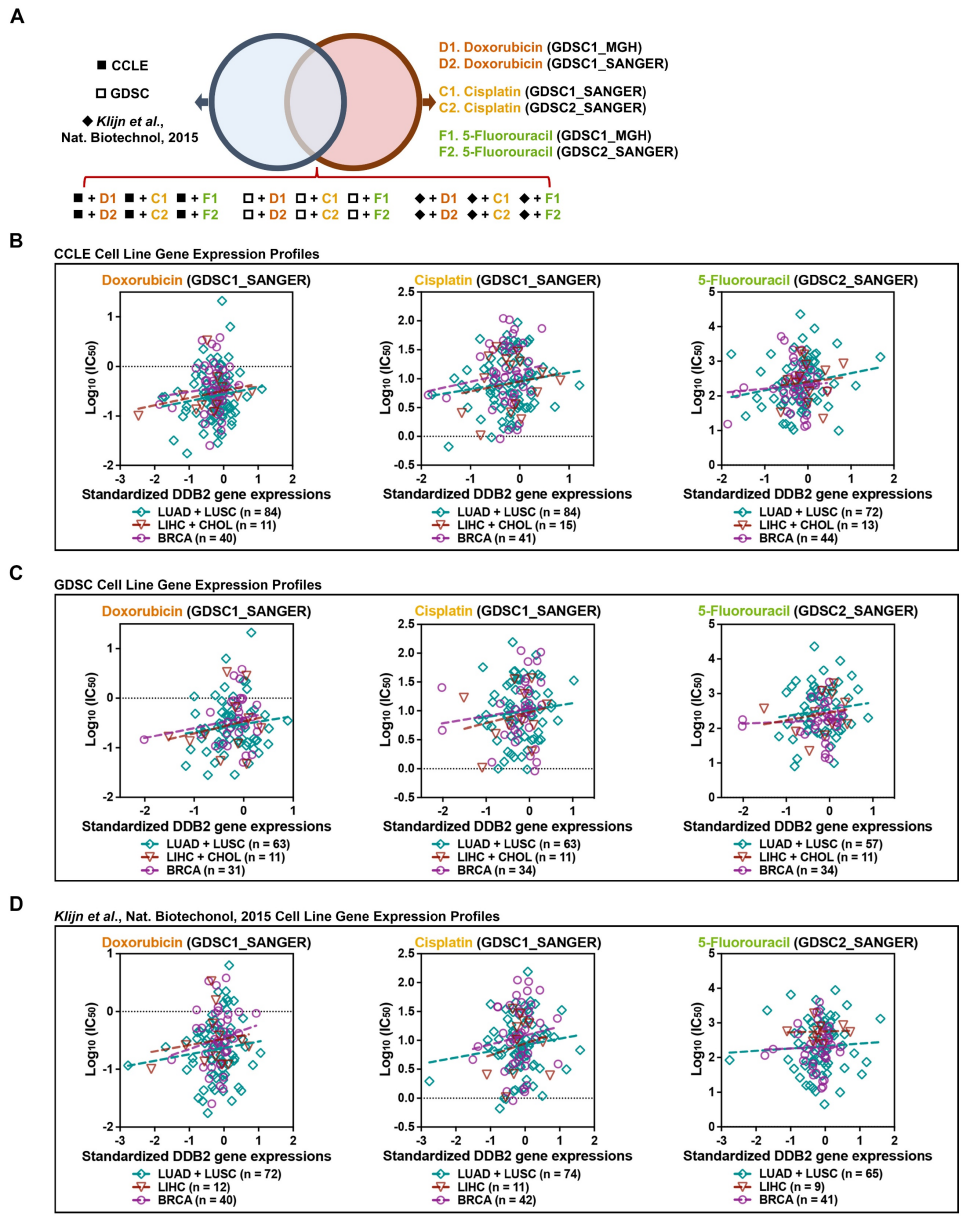
** Higher expression of DDB2 is positively correlated with chemoresistance and a poor response to treatments in solid tumors.** (A) The Venn diagram indicated that three cell line gene expression profiles from the pan-cancer analysis were intersected with six drug-sensitivity databases (http://www.cancerrxgene.org/, RRID: SCR_011956) from doxorubicin, cisplatin, and 5-FU treatments. (B-D) *In silico* analysis presented the correlation between DDB2 gene expression and the IC_50_ of LUAD, LUSC, LIHC, CHOL, and BRCA cell lines to doxorubicin, cisplatin, or 5-FU treatments.

To further investigate the role of DDB2 in mediating chemoresistance, we analyzed the correlation between DDB2 gene expression and sensitivity to chemotherapeutic agents in BRCA, LUAD, LUSC, LIHC, and CHOL cell lines (Genomics of Drug Sensitivity in Cancer) 35 using three independent datasets: the Cancer Cell Line Encyclopedia (CCLE) 36, GDSC 37, and *Klijn et al*. 38 (Figure 2A). DDB2 expression was positively correlated with the half-maximal inhibitory concentration (IC_50_) values for doxorubicin, cisplatin, and 5-FU across pan-cancer cell lines, indicating that higher DDB2 levels are associated with reduced drug sensitivity (Figure 2B-2D and Supplementary Figure S5A-S5C).

**Figure 3:**
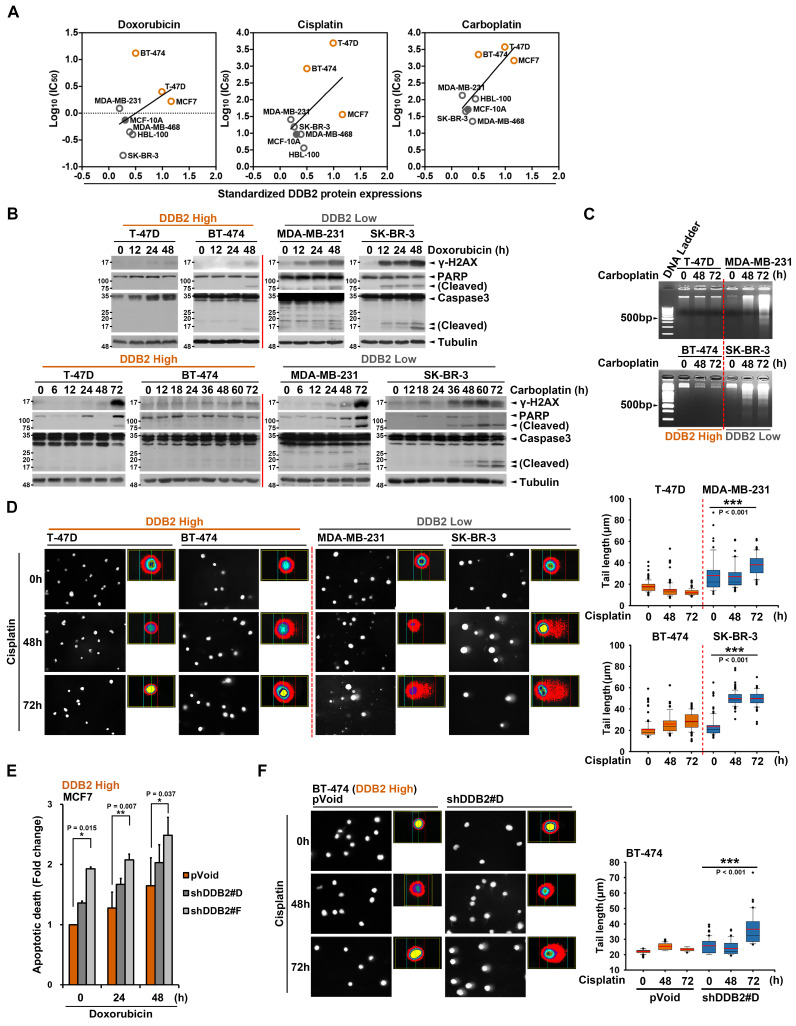
** DDB2 determines the chemosensitivity of breast cancer cells through DNA repair.** (A) The correlation between DDB2 protein levels and the IC_50_ of breast normal and breast cancer cell lines to doxorubicin, cisplatin, or carboplatin. (B) The DNA damage marker and apoptotic markers expression were examined in different breast cancer cell lines in response to doxorubicin (0.5 μM) or carboplatin (50 μM) in a time-dependent manner. (C) The DNA laddering assay was performed to evaluate the level of DNA damage and repair in different breast cancer cell lines in response to carboplatin (50 μM) in a time-dependent manner. (D) The comet assay was performed to examine the effect of cisplatin (50 μM) on DNA damage in various breast cancer cell lines. The tail moment and tail length index were calculated using Comet Assay III software. (E-F) Silencing DDB2 expression in high DDB2-expressing breast cancer cells enhanced doxorubicin (0.5 μM)- and cisplatin (50 μM)-induced higher apoptotic population in flow cytometry assay (E) and lower DNA repair capacity in comet assay (F). Representative data of three experiments was shown as the means ± SD. *p<0.05; **p<0.01; ***p<0.001 vs control group, Student's *t*-test.

We then selected BRCA as a primary model to validate the functional role of DDB2 in chemoresistance. Among ten breast cancer cell lines analyzed, MCF7, T-47D, and BT-474 cell lines exhibited the highest DDB2 protein levels (Supplementary Figure S6A). Importantly, DDB2 protein expression positively correlated with IC_50_ values for doxorubicin, cisplatin, and carboplatin (Figure 3A). Treatment with doxorubicin or carboplatin induced DNA damage, as evidenced by γ-H2AX expression, and triggered apoptosis, as shown by cleaved PARP and Caspase3, in low DDB2-expressing cancer cells (e.g., MDA-MB-231, SK-BR-3, MDA-MB-468). In contrast, these effects were markedly attenuated in high DDB2-expressing cancer cells (e.g., T-47D, BT-474, MCF7) (Figure 3B and Supplementary Figure S6B). Consistent with these findings, DNA laddering (Figure 3C) and increased tail moments in comet assays (Figure 3D and Supplementary Figure S6C) were observed following platinum-based chemotherapy only in low DDB2-expressing cells, suggesting that high DDB2 levels facilitate DNA repair and thereby mitigate DNA damage and apoptosis.

To further validate the causal role of DDB2 in chemoresistance, we silenced DDB2 expression using shRNA in high-expressing breast cancer cell lines (T-47D, BT-474, and MCF7). Knockdown of DDB2 significantly reduced the IC_50_ values for doxorubicin and carboplatin (Supplementary Figure S6D), enhanced chemotherapy-induced apoptotic responses, as shown by increased Annexin V-positive cells in flow cytometry (Figure 3E and Supplementary Figure S6E), elevated PARP and Caspase3 cleavage (Supplementary Figure S6F), and pronounced DNA damage in comet assays (Figure 3F). Together, these findings indicate the critical role of DDB2 in promoting chemoresistance through enhanced DNA repair and suppression of DNA damage-induced apoptosis in breast cancer cells.

### Identification of lapatinib as a potential disruptor of DDB2/DNA complex

**Figure 4:**
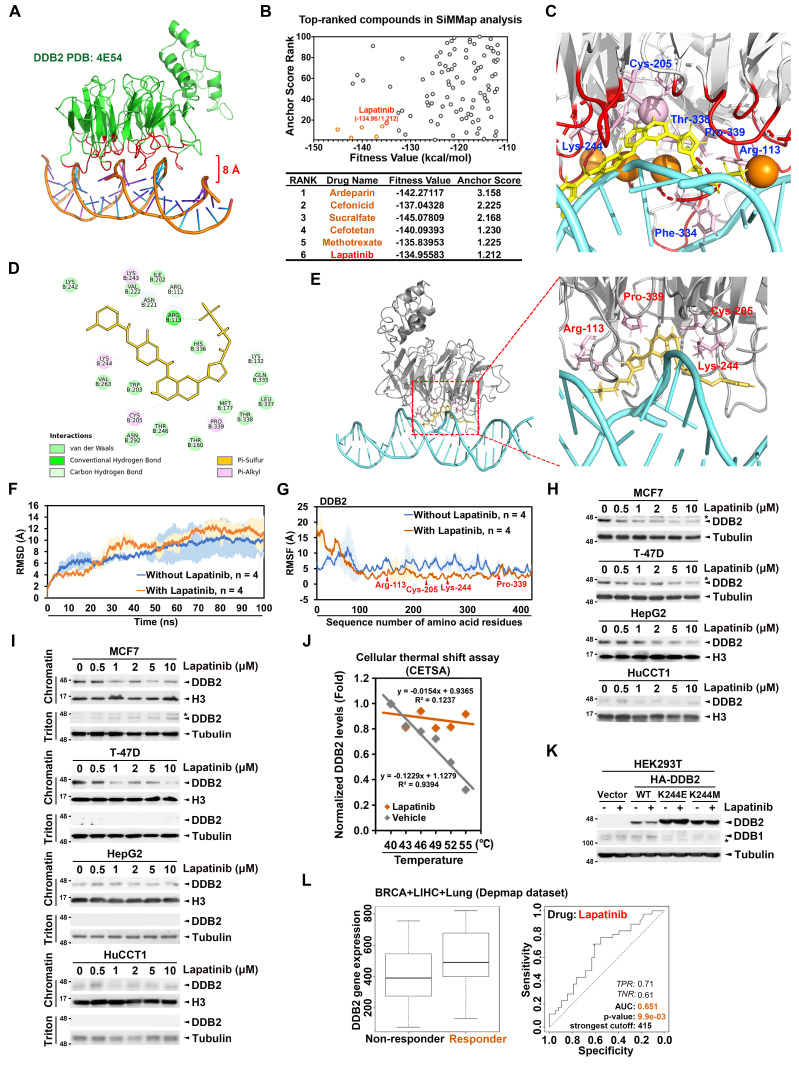
** Lapatinib is a potential disruptor of the DDB2/DNA complex.** (A) The structure of DDB2 protein (green)/DNA (orange) complex (4E54) was acquired from RCSB PDB (http://www.rcsb.org/#Category-welcome, RRID:SCR_012820), and the DNA-binding region (red) was defined as the residues which distance to DNA was within 8Å. (B) The top 100 ligands of docking results from the GEMDOCK program were analyzed for the compound moieties by a web server, SiMMap analysis (http://simmap.life.nctu.edu.tw), and re-rank by the Anchor Score. (C) The results utilized the SiMMap analysis presenting lapatinib (yellow) interacting with 6 amino acids (blue and pink) of the DNA-binding loop of DDB2. (D) The interacting analysis of lapatinib with the DNA-binding region of DDB2 by BIOVIA Discovery Studio software (http://accelrys.com/products/collaborative-science/biovia-discovery-studio/, RRID:SCR_015651). (E) The lapatinib (yellow)-DDB2 (gray) docking results showed in 3D structure and presented 4 amino acids (red and pink) of DDB2 to interact with lapatinib by PyMOL software (http://www.pymol.org/, RRID:SCR_000305). (F-G) The RMSD (F) and RMSF (G) results were analyzed from the MD simulations of lapatinib/DDB2 complex. The results are presented as the average value from four independent MD stimulations conducted over time. (H-I) The high DDB2-expressing cancer cells (MCF7, T-47D, HepG2, and HuCCT1) were treated with lapatinib in a dose-dependent manner for 48 hours, and the cell lysates were collected (H) or separated into chromatin-bound and chromatin-free fractions by triton extraction assay (I). (J) CETSA was used to detect the direct interaction between lapatinib (50 μM) and DDB2 protein. (K) The lysine mutations of DDB2 maintained the protein stability in lapatinib (1 μM) treatments. (L) ROC Plotter was subjected to validate the association of DDB2 expression with the response to lapatinib in breast, liver, and lung cancer and indicated DDB2 as a biomarker for lapatinib responsiveness.

To identify potential chemosensitizers that target DDB2/DNA complex, we performed an *in silico* screening of over one thousand FDA-approved compounds, aiming to predict candidate molecules capable of binding to the DNA-binding region (8 Å distance to the DNA strand) of the DDB2 protein (PDB ID: 4E54) 39 (Figure 4A). Molecular docking was performed using iGEMDOCK v2.1 26, 27 and based on the docking fitness values and anchor scores derived from the SiMMap analysis 28, the top ten compounds with the highest predicted binding affinity to the DNA-binding region of DDB2 were identified (Figure 4B). Among these candidates, lapatinib, a dual EGFR/HER2 tyrosine kinase inhibitor approved for the treatment of HER2-positive metastatic breast cancer 24, showed a high binding energy of -134.956 kcal/mol and an anchor score of 1.212. Lapatinib was predicted to interact with the DNA-binding loop of DDB2 via six key residues: Arg-113, Cys-205, Lys-244, Phe-334, Thr-338, and Pro-339 (labeled in blue) (Figure 4C). The docking results using BIOVIA Discovery Studio further validated this interaction, identifying Arg-112, Arg-113, Lys-132, Cys-205, Asn-221, Lys-243, Lys-244, and Pro-339 as the critical residues contributing to the lapatinib-DDB2 interaction (Figure 4D). Visualization using PyMOL confirmed that Arg-113, Cys-205, Lys-244, and Pro-339 (labeled in red) were consistently involved in stabilizing the lapatinib-DDB2 complex across modeling platforms (Figure 4E).

In addition, we have included a comparison of lapatinib's binding energy to DDB2 versus its known target EGFR tyrosine kinase domain. Molecular docking analyses using BIOVIA Discovery Studio showed that lapatinib binds to DDB2 with a predicted binding energy of -37.0256 kcal/mol, which is slightly lower than but comparable to its binding energies for EGFR (-72.2448 kcal/mol) (Supplementary Figure S7A). Compared to other EGFR tyrosine kinase inhibitors (TKIs), lapatinib exhibited the most favorable binding energy to DDB2, while others showed weaker or less stable interactions (Supplementary Figure S7B). These findings suggest that DDB2 binding may be preferential to lapatinib, supporting its repurposing as a DDB2-targeting agent. To provide evidence that lapatinib stably interacts with the DNA-binding region of DDB2, molecular dynamics (MD) simulations were conducted. During a 100-nanoseconds simulation, as the Root Mean Square Deviation (RMSD) values plateaued, the DDB2-DNA complex exhibited greater movement in the presence of lapatinib (Figure 4F). This increased motion may be attributed to the higher flexibility of the N-terminus of the DDB2 structure, as indicated by a higher Root Mean Square Fluctuation (RMSF) value. Meanwhile, the regions interacting with lapatinib, including Arg113, Cys205, Lys244, and Pro339, became more rigid and locked in a conformation that prevents proper DNA recognition or repair function (Figure 4G). Consistently, DDB2 maintains its DNA-associated structure throughout the simulation (Supplementary Video 1). However, lapatinib disrupts the DNA association and destabilizes the N-terminal region of the DDB2 protein (Supplementary Video 2). These findings suggest that lapatinib binds to DDB2 and restricts the conformational flexibility required for efficient DNA interaction, thereby potentially impairing its DNA-binding function.

Lapatinib treatment led to a dose-dependent reduction in DDB2 protein levels (Figure 4H and Supplementary Figure S7C) and a decrease in its chromatin-binding affinity (Figure 4I) in high DDB2-expressing breast (MCF7, T-47D), liver (HepG2), and cholangiocarcinoma (HuCCT1, KKU-100, and SNU-1196) cancer cell lines (Supplementary Figure S6A and S7D). A cellular thermal shift assay (CETSA) 40 further supported the direct binding between lapatinib and DDB2 protein *in vitro* (Figure 4J and Supplementary Figure S7E). Lys-244 (K244), a potential lapatinib-interacting residue, is essential for nucleotide binding and DDB2-mediated DNA repair 39. K244 mutations are associated with impaired DNA repair and Xeroderma Pigmentosum (XP) 39, 41. Importantly, the substitution of K244 with glutamate (K244E) or methionine (K244M) abolished the lapatinib-induced downregulation of DDB2, suggesting the critical role of this residue in mediating the drug's effect (Figure 4K). Finally, analysis using the ROC Plotter tool revealed that DDB2 expression was higher in tumor samples from patients who responded to lapatinib, suggesting that DDB2 may serve as a predictive biomarker for lapatinib responsiveness (Figure 4L). Collectively, these findings identify DDB2 as a novel molecular target of lapatinib and demonstrate that lapatinib can disrupt the DNA-binding capacity of DDB2, promote its degradation, and enhance chemosensitivity in cancers with high DDB2 expression.

### Synergistic enhancement of doxorubicin efficacy by lapatinib

Doxorubicin and platinum are widely used across multiple cancer types, yet the development of drug resistance often limits their clinical efficacy. Targeting DNA repair pathways has emerged as a promising strategy to overcome this challenge 42. Given our findings that lapatinib disrupts the DNA-binding activity of DDB2, we hypothesized that co-treatment with lapatinib could enhance the sensitivity of high DDB2-expressing cancer cells to doxorubicin.

**Figure 5:**
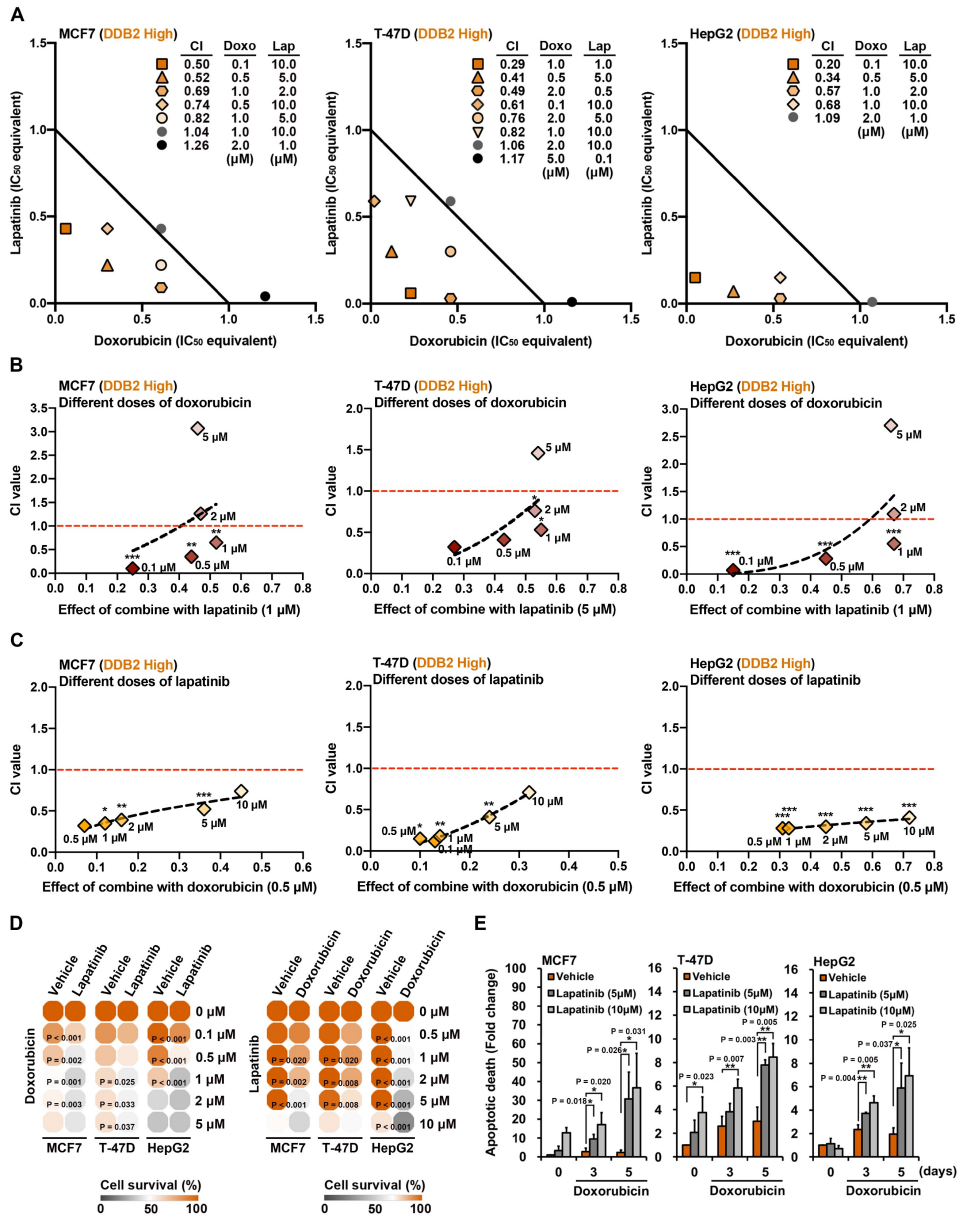
** Lapatinib enhances doxorubicin-induced cytotoxicity in high DDB2-expressing cancer cells.** (A) Isobologram analysis presented the synergistic effects of different dosages of lapatinib and doxorubicin and the calculated CI value. (B-C) The trends presented the combination effects between lapatinib and doxorubicin and CI values. (D) The survival rate was measured by using MTT assays and the heatmap was created by Morpheus-Broad Institute. (E) Cancer cell lines were treated with doxorubicin (0.2 μM) and/or lapatinib in a time-dependent manner, and apoptotic death was measured by using flow cytometry assays. Data was shown as the means ± SD. *p<0.05; **p<0.01; ***p<0.001 vs control group, Student's *t*-test.

To assess potential synergism, we performed an isobologram-based CI analysis using the IC_50_ values of doxorubicin and lapatinib, either as single agents or in combination 43. In high DDB2-expressing cancer cells (MCF7, T-47D, and HepG2), the combination treatment showed a CI<1, indicating synergistic inhibition of cell viability (Figure 5A). Synergism was observed when lapatinib was used at a fixed dose with decreasing concentrations of doxorubicin (Figure 5B) and vice versa (Figure 5C), suggesting that co-treatment could achieve therapeutic efficacy even at reduced doses of each drug.

Further validation using MTT assays demonstrated that the lapatinib-doxorubicin combination significantly decreased cell viability compared to single-agent treatments, as visualized by a heatmap of viability changes (Figure 5D). A similar result was observed in HuCCT1 cancer cells, which exhibited enhanced sensitivity to cisplatin and doxorubicin when combined with lapatinib (Supplementary Figure S8A). Additionally, the combination treatment increased the proportion of apoptotic cells in a time-dependent manner in high DDB2-expressing cell lines (Figure 5E and Supplementary Figure S8B), reinforcing the notion that lapatinib enhances cytotoxicity of chemotherapy through mechanisms beyond EGFR/HER2 inhibition, likely via disruption of DDB2-mediated DNA repair.

**Figure 6:**
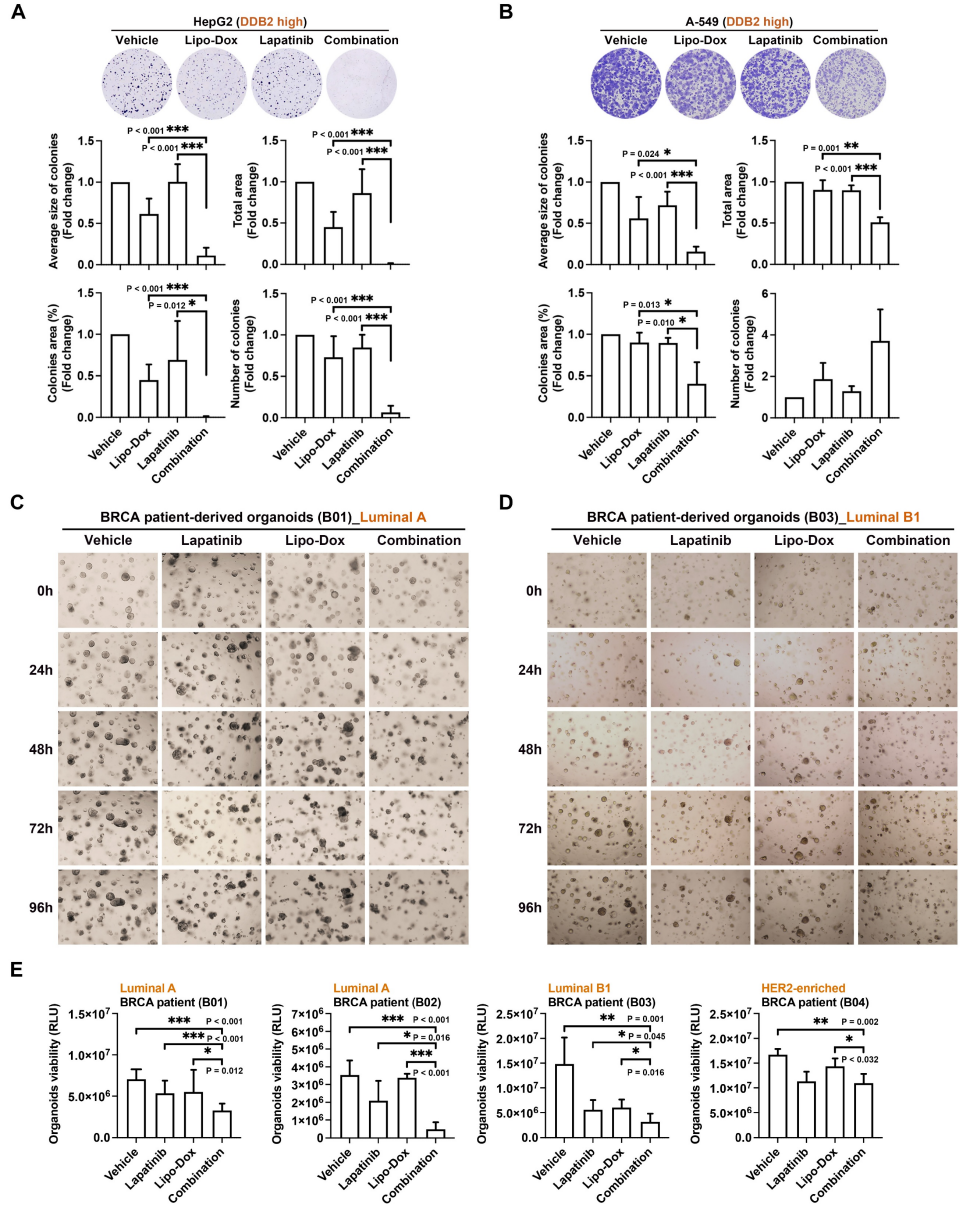
** Lapatinib enhances the anti-proliferative activity of Lipo-Dox in patient-derived organoids.** (A-B) The clonogenic assay was used to analyze the cell proliferation of HepG2 and A-549 cancer cells with indicated treatments with Lipo-Dox (0.2 μM) and/or lapatinib (1 μM) for 7 days. Quantitation data was calculated with ImageJ software. (C-E) The synergistic effect of Lipo-Dox (0.2 μM) and lapatinib (1 μM) was analyzed in BRCA-derived organoids. The viability of the organoids was quantified using the CellTiter-Glo 3D Cell Viability Assay. Data was shown as the means ± SD. *p<0.05; **p<0.01; ***p<0.001 vs control group, Student's *t*-test.

To explore its clinical applicability, we evaluated the effect of combining lapatinib with Lipo-Dox, a liposomal formulation of doxorubicin approved for use in a range of cancers. Colony formation assays revealed that the combination of 1 μM lapatinib with Lipo-Dox significantly suppressed clonogenic growth in high DDB2-expressing HepG2, A-549, and CL1-0 cancer cells (Figures 6A, 6B, and Supplementary Figure S9A). Quantitative analysis revealed a significant reduction in average colony size, colony area, and total growth area in the combination group compared to the vehicle and monotherapy groups. Notably, lapatinib enhanced the sensitivity of four tumor organoids derived from different BRCA cancer patients (2 Luminal A, 1 Luminal B1, and 1 HER2-enriched) to Lipo-Dox (Figure 6C, 6D, and 6E; Supplementary Figure S9B and S9C). Collectively, these findings demonstrate that lapatinib enhances the therapeutic efficacy of doxorubicin and Lipo-Dox in DDB2-high cancers. This combinatorial approach holds strong promise for improving treatment outcomes and reducing chemotherapy-related toxicity by enabling lower doses of drugs.

### Lapatinib-doxorubicin co-treatment inhibits chromatin-binding and induces protein degradation of DDB2

**Figure 7:**
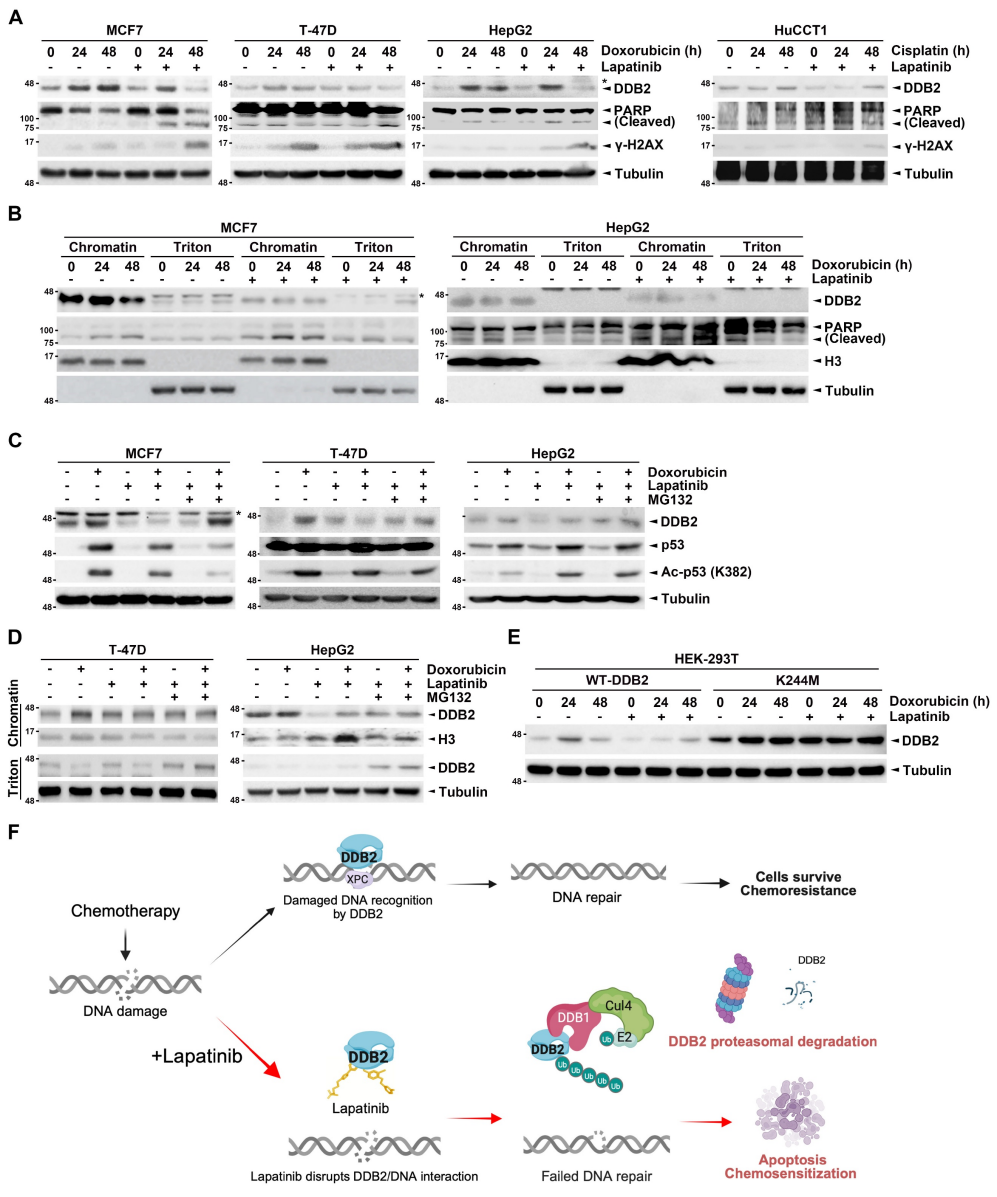
** Lapatinib inhibits the chromatin-binding activity of DDB2 and elicits its proteasomal degradation.** (A-B) High DDB2-expressing cancer cells were treated with doxorubicin (0.2 μM) or cisplatin (25 μM), either alone or in combination with lapatinib (1 μM) in a time-dependent manner. Total lysates (A) and chromatin-bound and chromatin-free fractions (B) were prepared by RIPA buffer or triton extraction assay followed by western blot assay with indicated antibodies. (C-D) Cancer cells with high DDB2 expression were treated with doxorubicin (0.2 μM), either alone or in combination with lapatinib (1 μM) and MG132 (10 μM), for 24 hours. Total cell lysates (C) or the lysates from triton extraction assay (D) were subjected to western blot analysis. (E) HEK293T cells were transfected with WT-DDB2 and MutDDB2 and treated with doxorubicin (0.2 μM) alone or in combination with doxorubicin (0.2 μM) and lapatinib (1 μM) followed by western blot analysis. (F) The proposed model illustrates that the combination treatment with lapatinib can prevent DDB2 from recognizing the site of DNA damage caused by chemotherapy, thereby impairing NER efficacy and overcoming chemoresistance in cancer cells.

Given the established role of proteasomal degradation in regulating DDB2 protein stability, we next investigated whether co-treatment with lapatinib and chemotherapy promotes proteasomal degradation of DDB2. As shown in Figure 7A and Supplementary Figure S9D, doxorubicin or cisplatin alone led to an upregulation of DDB2 expression, likely as a compensatory DNA repair response. However, co-treatment with lapatinib resulted in a time-dependent decrease in DDB2 protein levels, accompanied by elevated expression of γ-H2AX and cleaved PARP, indicating enhanced DNA damage and apoptosis. These findings suggest that lapatinib enhances the cytotoxic effects of chemotherapy by suppressing DDB2.

Further analysis revealed a time-dependent reduction in chromatin-bound DDB2 following lapatinib-doxorubicin treatment (Figure 7B), suggesting that impaired DNA binding is a key mechanism of DDB2 inactivation. To confirm whether the observed DDB2 degradation was mediated by the proteasome 44, high DDB2-expressing cancer cells were pre-treated with the proteasome inhibitor MG132. Notably, MG132 rescued DDB2 protein levels, which were diminished by the combination treatment (Figure 7C).

However, MG132 did not restore the chromatin-bound fraction of DDB2; instead, it led to an increase in the triton-soluble (non-chromatin-bound) pool of DDB2 in cells treated with both lapatinib and doxorubicin (Figure 7D). This finding indicates that lapatinib disrupts DDB2's chromatin-binding capacity independently of its degradation, and that the inhibition of DNA binding precedes proteasomal targeting. To further confirm that lapatinib interferes with DNA-binding via direct interaction with the DDB2 DNA-interacting domain, we assessed the impact of the K244M mutant, which abrogates DDB2's DNA recognition activity. Consistent with the earlier structural predictions, the K244M mutation prevented DDB2 degradation induced by lapatinib in the presence of doxorubicin (Figure 7E), supporting the functional importance of this residue in mediating lapatinib's effect.

Collectively, our findings indicate DDB2 as a critical mediator of chemoresistance through its role in facilitating DNA repair and suppressing apoptosis following chemotherapy in breast cancer, lung cancer, liver cancer, and cholangiocarcinoma. Elevated DDB2 expression correlates with poor therapeutic outcomes and reduced sensitivity to DNA-damaging agents. Importantly, we identify lapatinib, beyond its established EGFR/HER2 inhibitory function, as a novel disruptor of DDB2's DNA-binding activity, capable of enhancing chemosensitivity by impairing DNA repair and promoting proteasomal degradation of DDB2 (Figure 7F) (https://BioRender.com/h39jluk). These results highlight a previously unrecognized mechanism of lapatinib action and support its repurposing as a DDB2-targeted chemosensitizer. Targeting DDB2 with lapatinib represents a promising therapeutic strategy to overcome chemoresistance and improve the efficacy of DNA-damaging chemotherapies across multiple cancer types.

## Discussion

Chemoresistance remains a significant obstacle in the treatment of solid tumors, driven in part by the enhanced ability of cancer cells to repair DNA damage and evade apoptosis. In this study, we identify DDB2 as a key player in chemoresistance across multiple cancer types, including breast cancer, liver cancer, lung cancer, and cholangiocarcinoma. Through its roles in NER and HR, DDB2 promotes DNA repair following genotoxic stress, enabling cancer cells to survive treatment with DNA-damaging agents such as doxorubicin 17, platinum compounds 45, PARP inhibitors 16, or radiotherapy 46 in breast, colorectal, and pancreatic cancers, respectively. In these studies, elevated DDB2 expression was associated with reduced chemosensitivity and poorer prognosis, highlighting its potential as both a biomarker and therapeutic target. While the ROC analysis highlights DDB2's potential as a predictive biomarker, the retrospective nature of the datasets introduces limitations such as treatment heterogeneity and selection bias. Prospective validation and protein-level correlation will be essential to confirm its clinical utility.

The functional role of DDB2 in cancer is complex and context-dependent. While our findings and several prior studies support an oncogenic role for DDB2, particularly in breast cancer 47, melanoma 48, endometrial 49, and ovarian cancer 50 where it promotes proliferation, survival, and resistance to chemotherapy, other reports describe tumor-suppressive functions of DDB2 in different settings. For example, DDB2 inhibits EMT in colorectal and pancreatic cancers 19, 23, and sensitizes ovarian cancer cells to cisplatin or 5-FU through Bcl-2 suppression 9, 51. Conversely, DDB2 knockdown has been shown to enhance cell proliferation in gastric cancer 22. These apparently contradictory roles may be attributed to differences in tissue type, p53 status 52, hormonal receptor expression 17, and the stage of cancer progression 53. DDB2's dual function, as a DNA repair protein and transcriptional regulator 48, likely contributes to its ability to act either as a tumor suppressor or promoter, depending on the cellular context.

A major finding of this study is the identification of lapatinib as a noncanonical inhibitor of DDB2. Although the clinical use of lapatinib is limited by the development of acquired resistance in breast cancers 54-56, we demonstrate that lapatinib binds directly to the DNA-binding region of DDB2, inhibits its chromatin association, and promotes proteasomal degradation, thereby enhancing the cytotoxic effects of DNA-damaging chemotherapy. According to a pharmacokinetic study, patients receiving 1500 mg lapatinib daily reached a mean Cmax of 1.314 µg/mL (approximately 2.26 µM) 57, indicating that the concentration of 1 µM used in this study is well within the achievable plasma range and supports the translational potential of targeting DDB2 with lapatinib. This repurposed function of lapatinib is particularly relevant given its established clinical safety and availability. In a Phase I clinical trial, lapatinib plus PARP inhibitor veliparib therapy has a manageable safety profile and promising antitumor activity in advanced TNBC 58, supporting further investigation into this strategy as a means to exploit synthetic lethality by co-targeting NER and other DDR pathways.

Unlike classical DNA repair inhibitors such as PARP inhibitors, which target BER, or ATR inhibitors, which disrupt the replication stress response and checkpoint control, lapatinib interferes with NER by disrupting the DDB2-DNA interaction. This distinct mechanism not only broadens the landscape of DNA repair-targeted therapies but also presents a non-redundant strategy that may complement existing inhibitors in combination regimens. Interestingly, emerging evidence from the literature suggests that lapatinib may also interfere with other DNA repair pathways, further supporting its role as a potential DNA repair suppressor. For example, lapatinib has been reported to impair HR through the inhibition of the EGFR/PARP complex in breast cancer cells 59. Additionally, it has been shown to enhance the DNA-damaging effects of radiation and chemotherapeutic agents by promoting oxidative stress and replication stress 60. Lapatinib has also been implicated in sensitizing cells to PARP inhibitors through the impairment of DNA damage response signaling 58. Moreover, the off-target effect of lapatinib can upregulate death receptor 4/5 through JNK/c-Jun activation, enhancing TRAIL-induced apoptosis 61. These observations, together with our findings, suggest that lapatinib may have broader applications as a chemosensitizer targeting DNA repair pathways, extending beyond its conventional role in EGFR/HER2 inhibition. Importantly, the K244M mutation in DDB2, which disrupts its DNA recognition capability, abolished the effects of lapatinib, highlighting the specificity of this interaction. Our study provides a strong rationale for using lapatinib to target DDB2-dependent DNA repair mechanisms, particularly in tumors with high DDB2 expression that are resistant to conventional chemotherapy. However, enhancement of NF-κB activation and COX-2 expression in a kinase-inactive EGFR-dependent manner may limit the use of lapatinib 55, 62. Therefore, in future clinical applications, lapatinib may be combined with other novel therapeutics with different mechanisms of action, such as RNA-based drugs or specific small-molecule inhibitors, to target DNA repair gene expression at the transcriptional level. This combined approach could complement the protein-level action of lapatinib, offering a dual blockade from the transcriptional source to the protein endpoint, thereby broadening its therapeutic potential in cancer treatment.

Overall, this work not only reveals a previously unrecognized function of lapatinib in targeting DDB2 but also underscores the potential to repurpose lapatinib as a DNA repair-targeting agent. This approach could be particularly valuable in cancers that lack EGFR/HER2 amplification but are highly reliant on DNA repair for survival. Future directions include *in vivo* validation of this therapeutic strategy, the development of more selective DDB2 inhibitors, and the exploration of DDB2 as a predictive biomarker for treatment response.

## Supplementary Material

Supplementary figures.

Supplementary video 1: simulation of DDB2 without lapatinib, final.

Supplementary video 2: simulation of DDB2 with lapatinib, final.

## References

[1] Bray F, Laversanne M, Sung H, Ferlay J, Siegel RL, Soerjomataram I (2024). Global cancer statistics 2022: GLOBOCAN estimates of incidence and mortality worldwide for 36 cancers in 185 countries. CA Cancer J Clin.

[2] Anand U, Dey A, Chandel AKS, Sanyal R, Mishra A, Pandey DK (2023). Cancer chemotherapy and beyond: Current status, drug candidates, associated risks and progress in targeted therapeutics. Genes Dis.

[3] Harper JW, Elledge SJ (2007). The DNA damage response: ten years after. Mol Cell.

[4] Abad E, Graifer D, Lyakhovich A (2020). DNA damage response and resistance of cancer stem cells. Cancer Lett.

[5] Roos WP, Kaina B (2013). DNA damage-induced cell death: from specific DNA lesions to the DNA damage response and apoptosis. Cancer Lett.

[6] Hanawalt PC, Spivak G (2008). Transcription-coupled DNA repair: two decades of progress and surprises. Nat Rev Mol Cell Biol.

[7] Rocha JC, Busatto FF, de Souza LK, Saffi J (2016). Influence of nucleotide excision repair on mitoxantrone cytotoxicity. DNA Repair (Amst).

[8] Li LY, Guan YD, Chen XS, Yang JM, Cheng Y (2020). DNA Repair Pathways in Cancer Therapy and Resistance. Front Pharmacol.

[9] Bennett D, Itoh T (2008). The XPE gene of xeroderma pigmentosum, its product and biological roles. Adv Exp Med Biol.

[10] Scrima A, Konickova R, Czyzewski BK, Kawasaki Y, Jeffrey PD, Groisman R (2008). Structural basis of UV DNA-damage recognition by the DDB1-DDB2 complex. Cell.

[11] Barckhausen C, Roos WP, Naumann SC, Kaina B (2014). Malignant melanoma cells acquire resistance to DNA interstrand cross-linking chemotherapeutics by p53-triggered upregulation of DDB2/XPC-mediated DNA repair. Oncogene.

[12] Batista LF, Roos WP, Christmann M, Menck CF, Kaina B (2007). Differential sensitivity of malignant glioma cells to methylating and chloroethylating anticancer drugs: p53 determines the switch by regulating xpc, ddb2, and DNA double-strand breaks. Cancer Res.

[13] Yu F, Zheng S, Yu C, Gao S, Shen Z, Nar R (2025). KRAS mutants confer platinum resistance by regulating ALKBH5 posttranslational modifications in lung cancer. J Clin Invest.

[14] Bao N, Han J, Zhou H (2022). A protein with broad functions: damage-specific DNA-binding protein 2. Mol Biol Rep.

[15] Kattan Z, Marchal S, Brunner E, Ramacci C, Leroux A, Merlin JL (2008). Damaged DNA binding protein 2 plays a role in breast cancer cell growth. PLoS One.

[16] Zhao L, Si CS, Yu Y, Lu JW, Zhuang Y (2019). Depletion of DNA damage binding protein 2 sensitizes triple-negative breast cancer cells to poly ADP-ribose polymerase inhibition by destabilizing Rad51. Cancer Sci.

[17] He YH, Yeh MH, Chen HF, Wang TS, Wong RH, Wei YL (2021). ERalpha determines the chemo-resistant function of mutant p53 involving the switch between lincRNA-p21 and DDB2 expressions. Mol Ther Nucleic Acids.

[18] Huang HY, Chen CH, Cheng FJ, Wang BW, Tu CY, Chen YJ (2024). Incense-burning smoke ingredient Auramine enhances lincRNA-p21 expression for chemosensitization in p53-mutated non-small cell lung cancer. J Hazard Mater.

[19] Dardare J, Witz A, Betz M, Francois A, Meras M, Lamy L (2022). DDB2 represses epithelial-to-mesenchymal transition and sensitizes pancreatic ductal adenocarcinoma cells to chemotherapy. Front Oncol.

[20] Pavlopoulou A, Oktay Y, Vougas K, Louka M, Vorgias CE, Georgakilas AG (2016). Determinants of resistance to chemotherapy and ionizing radiation in breast cancer stem cells. Cancer Lett.

[21] Cui T, Srivastava AK, Han C, Wu D, Wani N, Liu L (2018). DDB2 represses ovarian cancer cell dedifferentiation by suppressing ALDH1A1. Cell Death Dis.

[22] Qiao S, Guo W, Liao L, Wang L, Wang Z, Zhang R (2015). DDB2 is involved in ubiquitination and degradation of PAQR3 and regulates tumorigenesis of gastric cancer cells. Biochem J.

[23] Roy N, Bommi PV, Bhat UG, Bhattacharjee S, Elangovan I, Li J (2013). DDB2 suppresses epithelial-to-mesenchymal transition in colon cancer. Cancer Res.

[24] Moreira C, Kaklamani V (2010). Lapatinib and breast cancer: current indications and outlook for the future. Expert Rev Anticancer Ther.

[25] Cheng FJ, Chen CH, Tsai WC, Wang BW, Yu MC, Hsia TC (2021). Cigarette smoke-induced LKB1/AMPK pathway deficiency reduces EGFR TKI sensitivity in NSCLC. Oncogene.

[26] Yang JM, Chen CC (2004). GEMDOCK: a generic evolutionary method for molecular docking. Proteins.

[27] Bitencourt-Ferreira G, de Azevedo WF Jr (2019). Docking with GemDock. Methods Mol Biol.

[28] Chen YF, Hsu KC, Lin SR, Wang WC, Huang YC, Yang JM (2010). SiMMap: a web server for inferring site-moiety map to recognize interaction preferences between protein pockets and compound moieties. Nucleic Acids Res.

[29] Sachs N, de Ligt J, Kopper O, Gogola E, Bounova G, Weeber F (2018). A Living Biobank of Breast Cancer Organoids Captures Disease Heterogeneity. Cell.

[30] Tibor Fekete J, Gyorffy B (2022). A unified platform enabling biomarker ranking and validation for 1562 drugs using transcriptomic data of 1250 cancer cell lines. Comput Struct Biotechnol J.

[31] Otília Menyhart WJK, Balázs Győrffy A gene set enrichment analysis for the cancer hallmarks. Journal of Pharmaceutical Analysis. 2024: 101065.

[32] Tang Z, Li C, Kang B, Gao G, Li C, Zhang Z (2017). GEPIA: a web server for cancer and normal gene expression profiling and interactive analyses. Nucleic Acids Res.

[34] Gyorffy B (2023). Discovery and ranking of the most robust prognostic biomarkers in serous ovarian cancer. Geroscience.

[35] Garnett MJ, Edelman EJ, Heidorn SJ, Greenman CD, Dastur A, Lau KW (2012). Systematic identification of genomic markers of drug sensitivity in cancer cells. Nature.

[36] Barretina J, Caponigro G, Stransky N, Venkatesan K, Margolin AA, Kim S (2012). The Cancer Cell Line Encyclopedia enables predictive modelling of anticancer drug sensitivity. Nature.

[37] Yang W, Soares J, Greninger P, Edelman EJ, Lightfoot H, Forbes S (2013). Genomics of Drug Sensitivity in Cancer (GDSC): a resource for therapeutic biomarker discovery in cancer cells. Nucleic Acids Res.

[38] Klijn C, Durinck S, Stawiski EW, Haverty PM, Jiang Z, Liu H (2015). A comprehensive transcriptional portrait of human cancer cell lines. Nat Biotechnol.

[39] Yeh JI, Levine AS, Du S, Chinte U, Ghodke H, Wang H (2012). Damaged DNA induced UV-damaged DNA-binding protein (UV-DDB) dimerization and its roles in chromatinized DNA repair. Proc Natl Acad Sci U S A.

[40] Jafari R, Almqvist H, Axelsson H, Ignatushchenko M, Lundback T, Nordlund P (2014). The cellular thermal shift assay for evaluating drug target interactions in cells. Nat Protoc.

[41] Matsuda N, Azuma K, Saijo M, Iemura S, Hioki Y, Natsume T (2005). DDB2, the xeroderma pigmentosum group E gene product, is directly ubiquitylated by Cullin 4A-based ubiquitin ligase complex. DNA Repair (Amst).

[42] Bowden NA (2014). Nucleotide excision repair: why is it not used to predict response to platinum-based chemotherapy?. Cancer Lett.

[43] Zhao L, Au JL, Wientjes MG (2010). Comparison of methods for evaluating drug-drug interaction. Front Biosci (Elite Ed).

[44] Motegi A, Murakawa Y, Takeda S (2009). The vital link between the ubiquitin-proteasome pathway and DNA repair: impact on cancer therapy. Cancer Lett.

[45] Slyskova J, Muniesa-Vargas A, da Silva IT, Drummond R, Park J, Hackes D (2023). Detection of oxaliplatin- and cisplatin-DNA lesions requires different global genome repair mechanisms that affect their clinical efficacy. NAR Cancer.

[46] Dardare J, Witz A, Betz M, Francois A, Lamy L, Husson M (2024). DDB2 expression lights the way for precision radiotherapy response in PDAC cells, with or without olaparib. Cell Death Discov.

[47] Matsumoto S, Fischer ES, Yasuda T, Dohmae N, Iwai S, Mori T (2015). Functional regulation of the DNA damage-recognition factor DDB2 by ubiquitination and interaction with xeroderma pigmentosum group C protein. Nucleic Acids Res.

[48] Zhang C, Li W, Liu L, Li M, Sun H, Zhang C (2024). DDB2 promotes melanoma cell growth by transcriptionally regulating the expression of KMT2A and predicts a poor prognosis. FASEB J.

[49] Hameed JSF, Devarajan A, M SD, Bhattacharyya A, Shirude MB, Dutta D (2023). PTEN-negative endometrial cancer cells protect their genome through enhanced DDB2 expression associated with augmented nucleotide excision repair. BMC Cancer.

[50] Wang G, Xu B, Yu X, Liu M, Wu T, Gao W (2024). LINC01320 facilitates cell proliferation and migration of ovarian cancer via regulating PURB/DDB2/NEDD4L/TGF-beta axis. Sci Rep.

[51] Park JS, Young Yoon S, Kim JM, Yeom YI, Kim YS, Kim NS (2004). Identification of novel genes associated with the response to 5-FU treatment in gastric cancer cell lines using a cDNA microarray. Cancer Lett.

[52] Krishnaraj J, Yamamoto T, Ohki R (2023). p53-Dependent Cytoprotective Mechanisms behind Resistance to Chemo-Radiotherapeutic Agents Used in Cancer Treatment. Cancers (Basel).

[53] Gilson P, Drouot G, Witz A, Merlin JL, Becuwe P, Harle A (2019). Emerging Roles of DDB2 in Cancer. Int J Mol Sci.

[54] Jabbarzadeh Kaboli P, Chen HF, Babaeizad A, Roustai Geraylow K, Yamaguchi H, Hung MC (2024). Unlocking c-MET: A comprehensive journey into targeted therapies for breast cancer. Cancer Lett.

[55] Hsia TC, Tu CY, Chen YJ, Wei YL, Yu MC, Hsu SC (2013). Lapatinib-mediated cyclooxygenase-2 expression via epidermal growth factor receptor/HuR interaction enhances the aggressiveness of triple-negative breast cancer cells. Mol Pharmacol.

[56] Hsiao YC, Yeh MH, Chen YJ, Liu JF, Tang CH, Huang WC (2015). Lapatinib increases motility of triple-negative breast cancer cells by decreasing miRNA-7 and inducing Raf-1/MAPK-dependent interleukin-6. Oncotarget.

[57] van der Noll R, Smit WM, Wymenga AN, Boss DS, Grob M, Huitema AD (2015). Phase I and pharmacological trial of lapatinib in combination with gemcitabine in patients with advanced breast cancer. Invest New Drugs.

[58] Stringer-Reasor EM, May JE, Olariu E, Caterinicchia V, Li Y, Chen D (2021). An open-label, pilot study of veliparib and lapatinib in patients with metastatic, triple-negative breast cancer. Breast Cancer Res.

[59] Nowsheen S, Cooper T, Stanley JA, Yang ES (2012). Synthetic lethal interactions between EGFR and PARP inhibition in human triple negative breast cancer cells. PLoS One.

[60] Yu T, Cho BJ, Choi EJ, Park JM, Kim DH, Kim IA (2016). Radiosensitizing effect of lapatinib in human epidermal growth factor receptor 2-positive breast cancer cells. Oncotarget.

[61] Dolloff NG, Mayes PA, Hart LS, Dicker DT, Humphreys R, El-Deiry WS (2011). Off-target lapatinib activity sensitizes colon cancer cells through TRAIL death receptor up-regulation. Sci Transl Med.

[62] Chen YJ, Yeh MH, Yu MC, Wei YL, Chen WS, Chen JY (2013). Lapatinib-induced NF-kappaB activation sensitizes triple-negative breast cancer cells to proteasome inhibitors. Breast Cancer Res.

