# Supplementary material for: Disrupting DDB2-DNA Interaction by Lapatinib Enhances Chemotherapy Sensitivity
Source: Int J Biol Sci. 2025 Sep 23;21(14):6007–24. doi: 10.7150/ijbs.116148 (PMC12594552; doi:10.7150/ijbs.116148)
Supplement: Supplementary file 1 — Supplementary figures. [file ijbsv21p6007s1.pdf]

Fig. S1

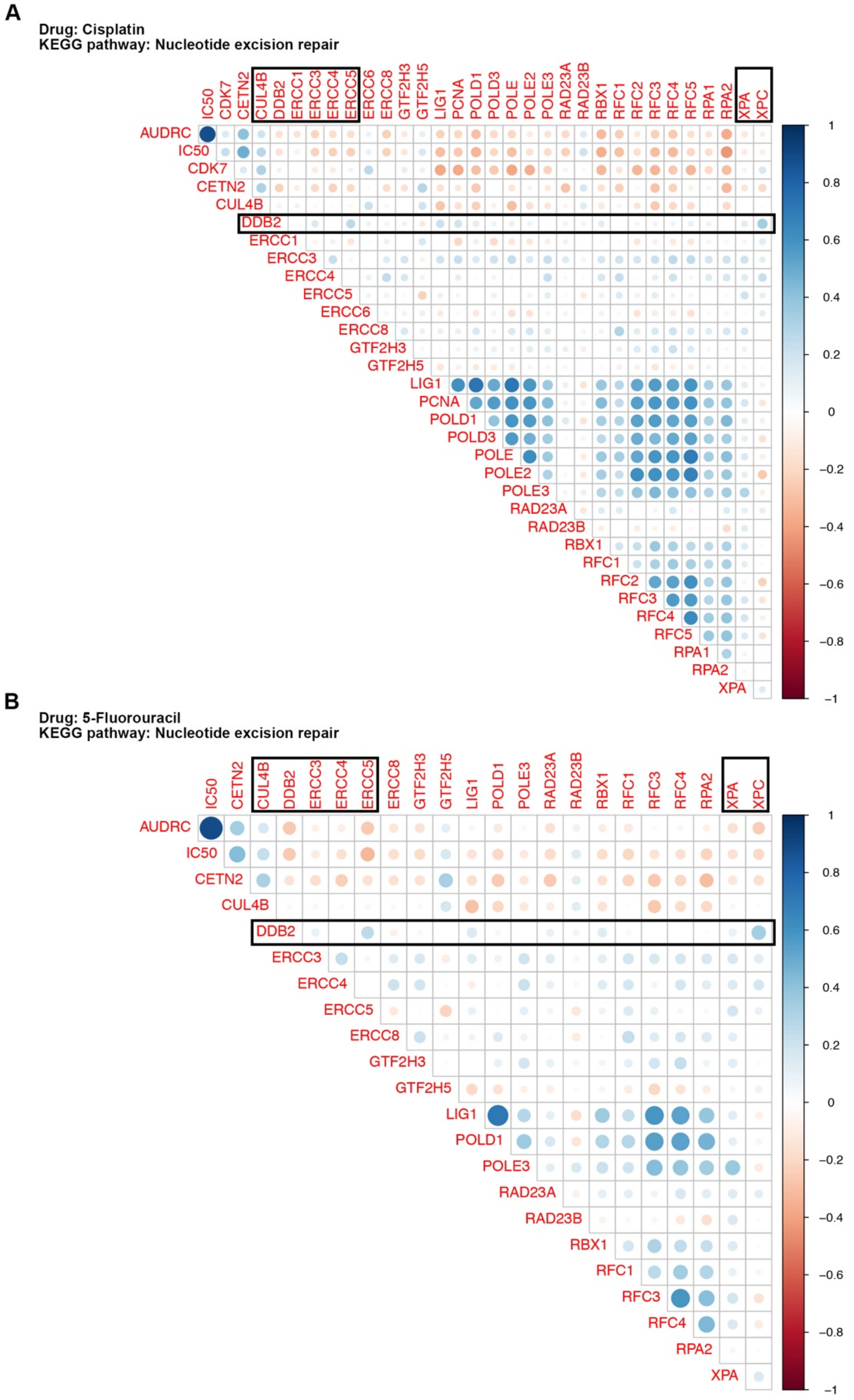

Figure S1. Gene correlation in nucleotide excision repair. (A-B) The correlation matrix chart analysis presented in NER with cisplatin (A), and with 5-FU (B).

Fig. S2

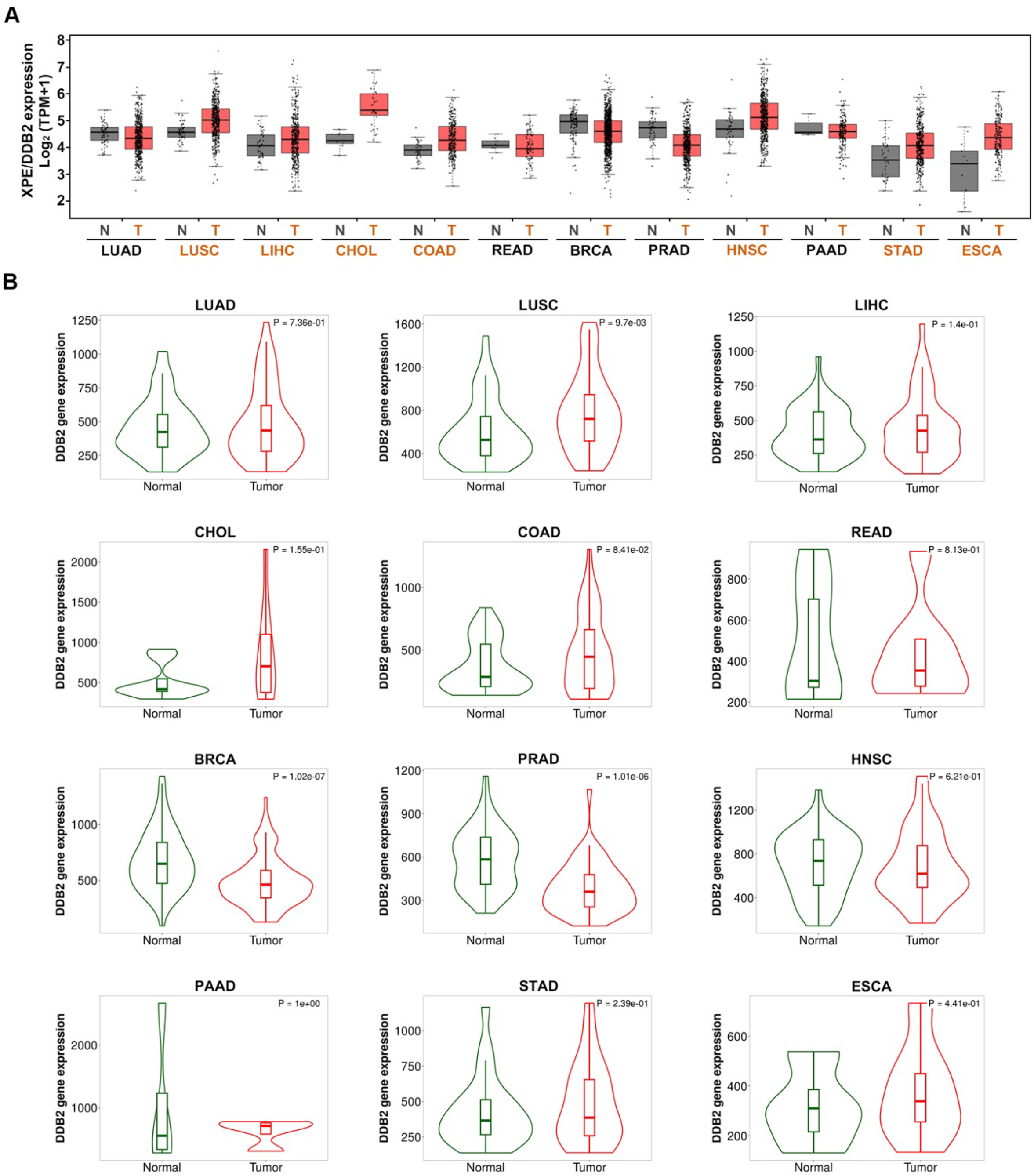

Figure S2. DDB2 expression profiles in pan-cancer analysis. (A-B) The box plot of DDB2 expression in each cancer type was analyzed from the pan-cancer databases, GEPIA (A) and TNMplot (https://tnmplot.com/analysis/) (B).

Fig. S3

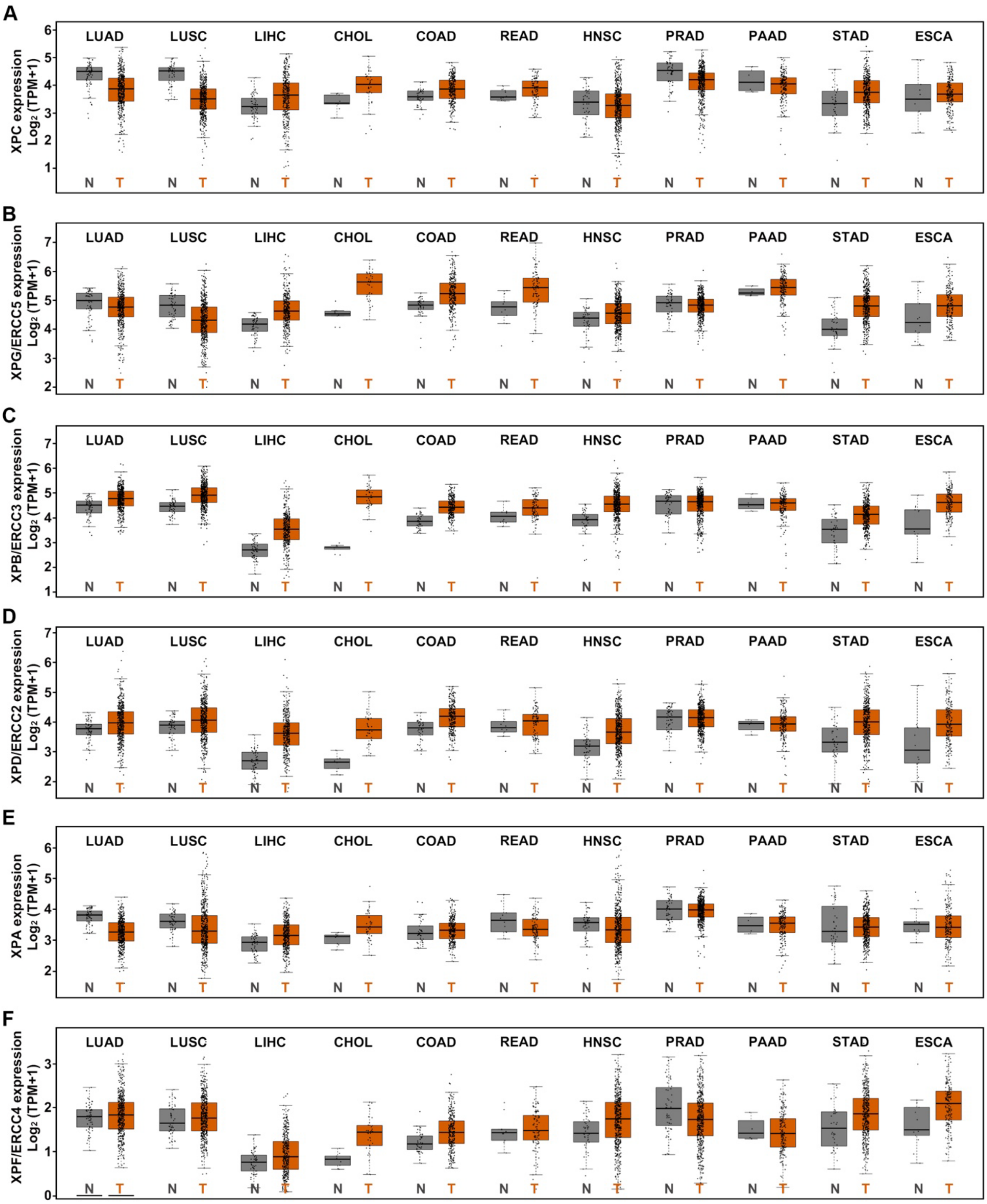

Figure S3. NER-related genes expression profiles in pan-cancer analysis. (A-F) The pan-cancer database, GEPIA, was used to analyze the levels of NER-related genes, XPC (A), XPG/ERCC5 (B), XPB/ERCC3 (C), XPD/ERCC2 (D), XPA (E), and XPF/ERCC4 (F) in each cancer type.

Fig. S4

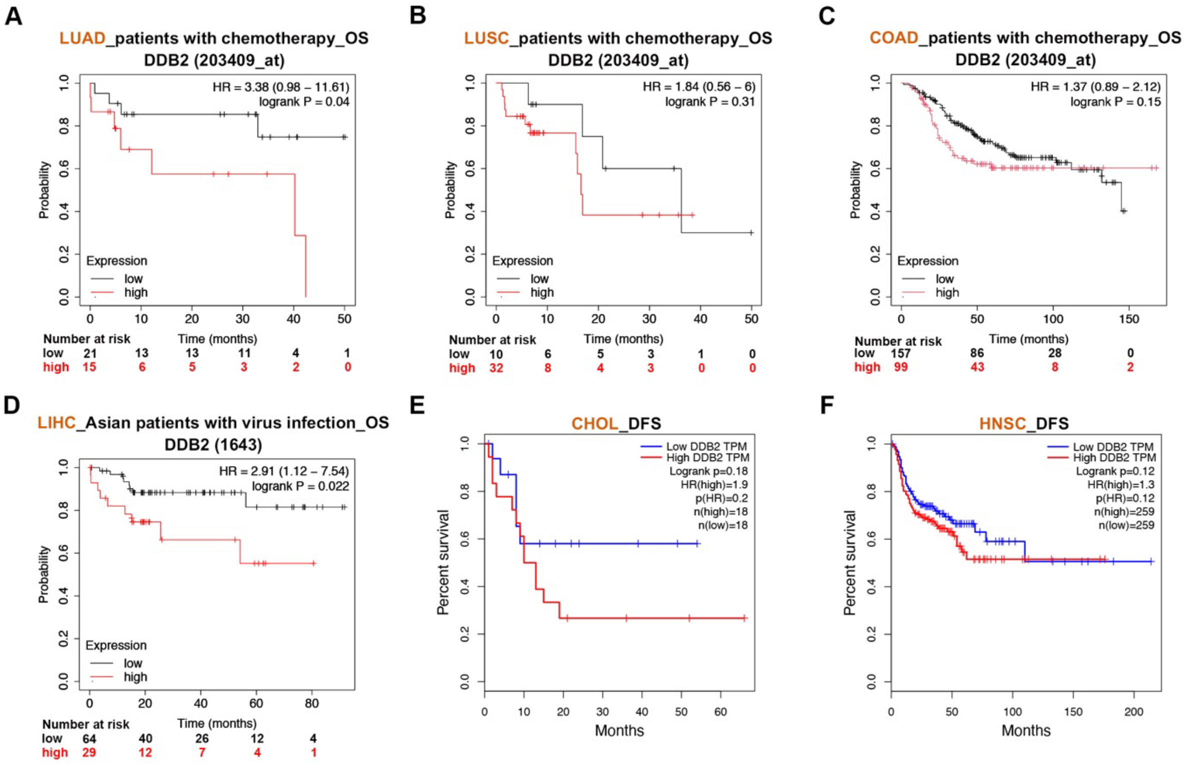

Figure S4. The survival in top cancer types. (A-F) Kaplan-Meier plotter (https://kmplot.com/analysis/, RRID: SCR_018753) was used to analyze the survival rates of top cancer types; LUAD patients received chemotherapy (A), LUSC patients received chemotherapy (B), COAD patients received chemotherapy (C), LIHC patients with virus infection (D), CHOL patients (E), and HNSC patients (F).

Fig. S5

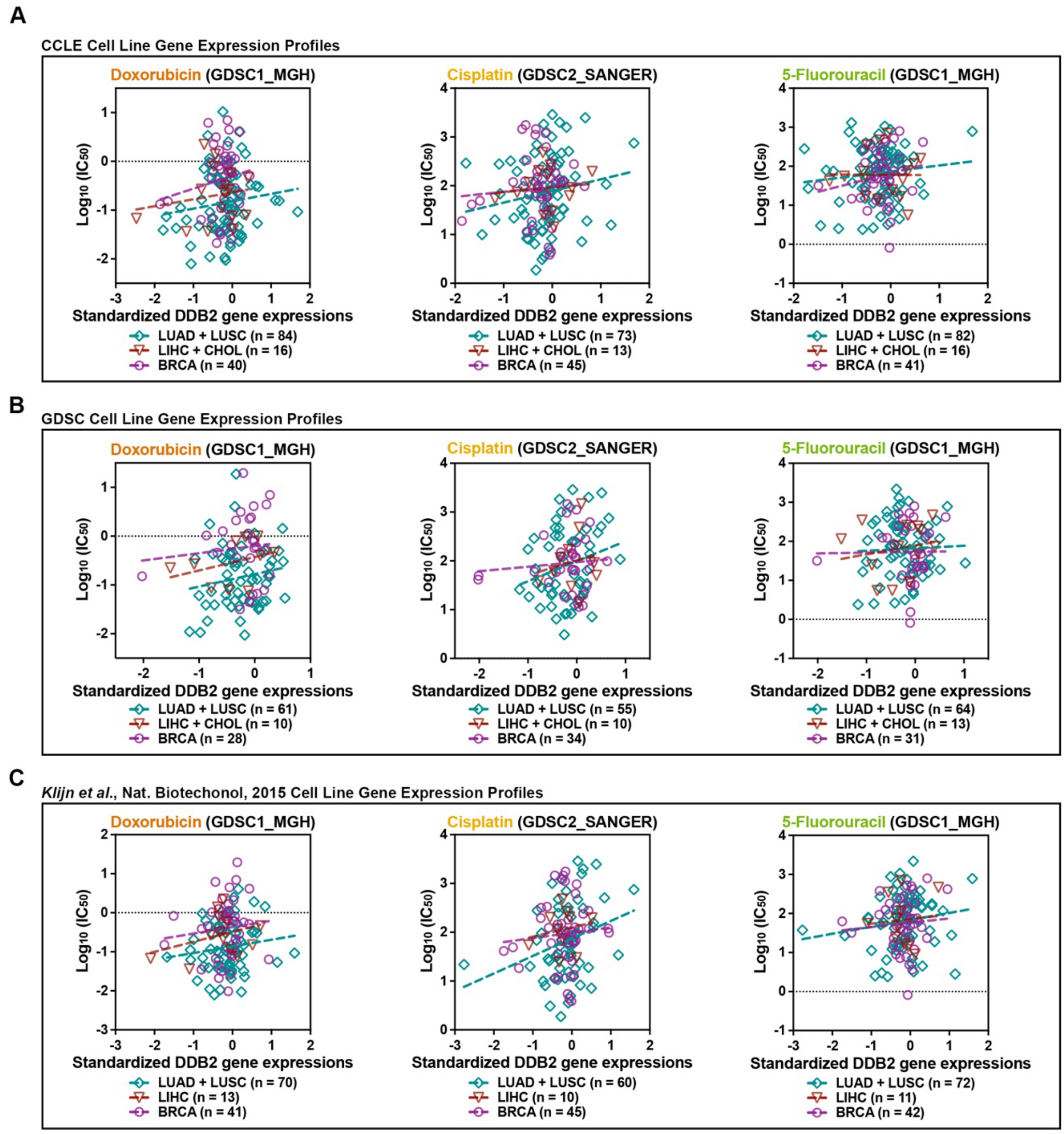

Figure S5. Correlation between DDB2 expression and chemosensitivity in solid tumors. (A-C) *In silico* analysis continued from Figure 2.

Fig. S6

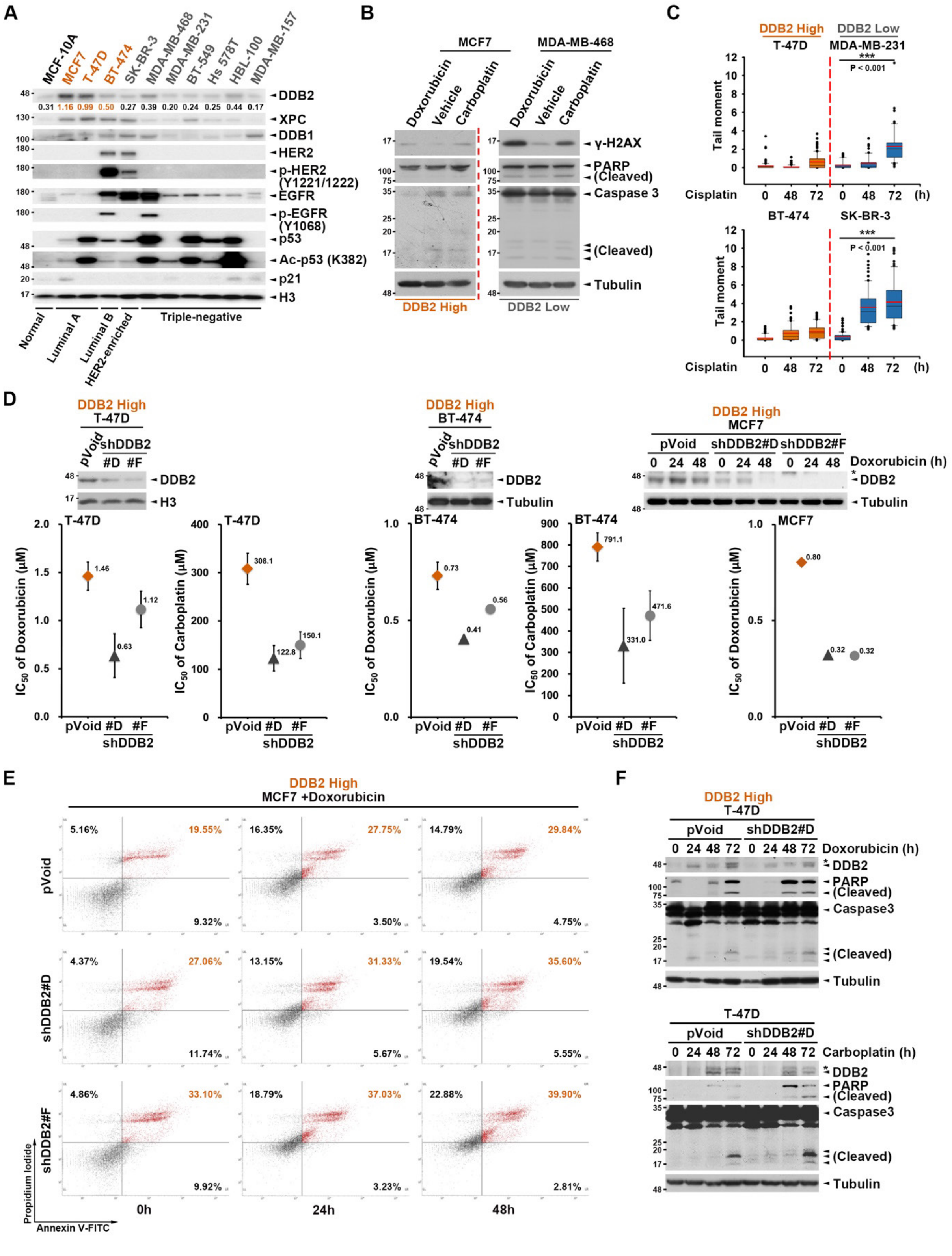

Figure S6. Silencing DDB2 expression enhanced chemosensitivity. (A) Basal protein levels of DDB2, p53, Ac-p53 (K382), p21, XPC (NER protein), DDB1 (NER protein), and other markers of breast cancer cells were examined by western blot assay. (B) DNA damage marker and apoptotic marker expression were examined in breast

cancer cell lines in response to doxorubicin (0.5 μM) or carboplatin (50 μM) for 48 and 72 hours, respectively. (C) Tail moment calculated from comet assay in various breast cancer cells treated with cisplatin (50 μM). (D-F) Knockdown of DDB2 expression by two independent shRNAs enhanced doxorubicin (0.5 μM)- and carboplatin (50 μM)-induced cell killing in MTT assay (D), increased apoptotic population in flow cytometry assay (F), and apoptotic markers were detected by western blot assay (D). Data was shown as the means ± SD. $^*p<0.05$; $^{**}p<0.01$; $^{***}p<0.001$ vs control group, Student's *t*-test.

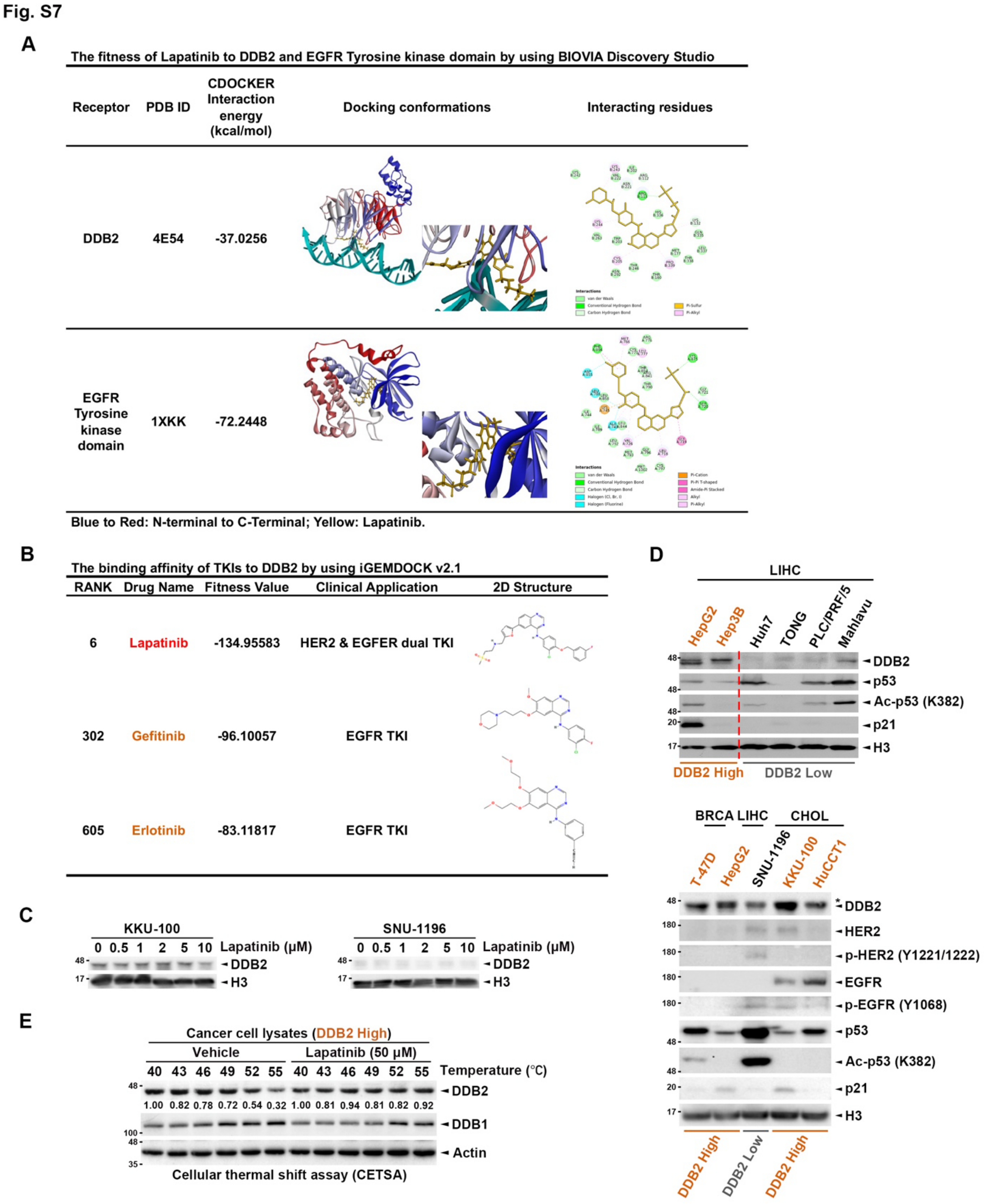

Figure S7. DDB2 is a potential target of lapatinib. (A) Docking results from the BIOVIA Discovery Studio system revealed that lapatinib binds to the DDB2 and EGFR tyrosine kinase domains. (B) The GEMDOCK program was used to analyze the affinity of TKIs for DDB2. (C) Cholangiocarcinoma cells (KKU-100 and SNU-1196) were treated with lapatinib in a dose-dependent manner for 48 hours. (D) DDB2 expression in various liver cancer and cholangiocarcinoma cell lines. (E) Raw data for Figure 4J.

Fig. S8

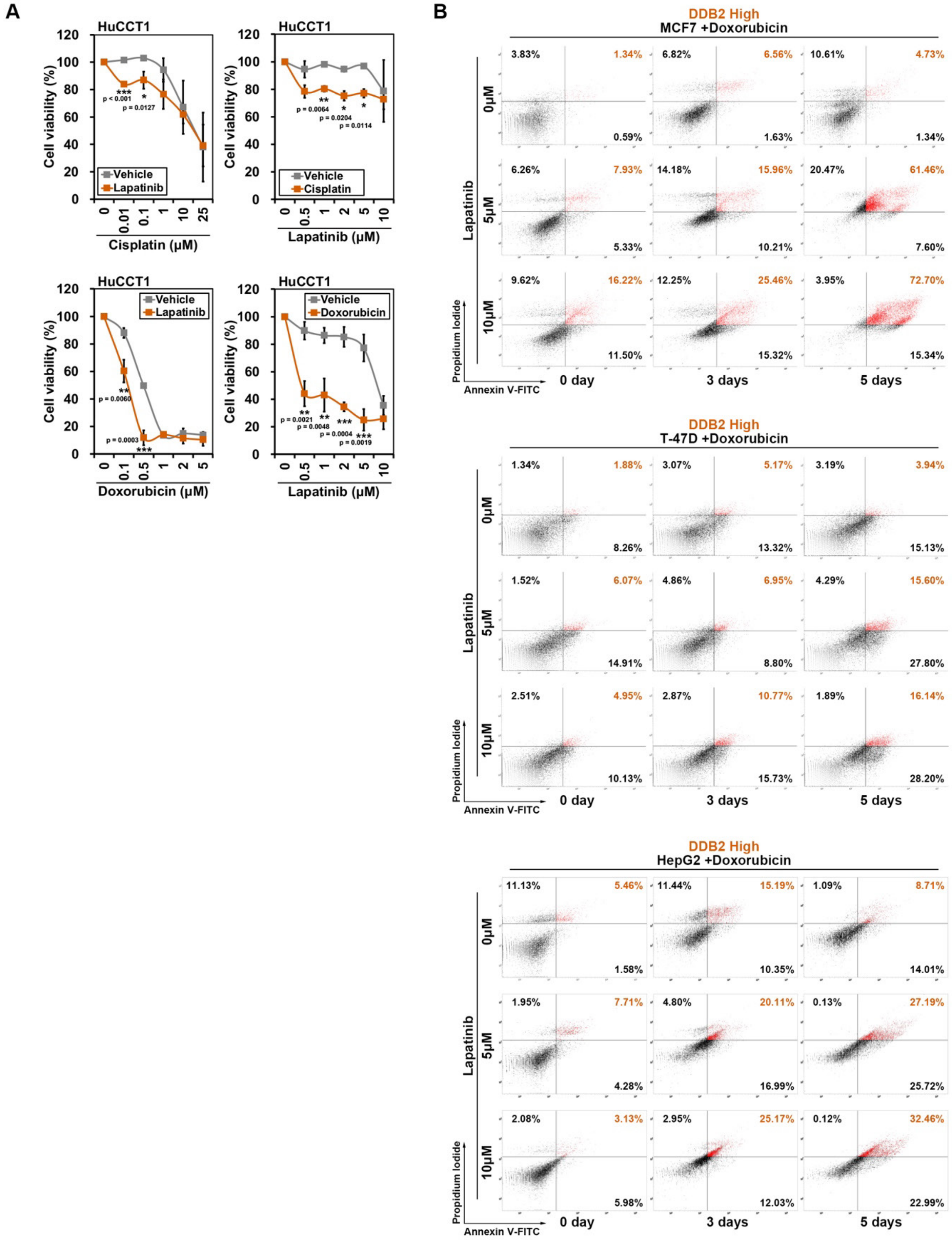

Figure S8. Lapatinib enhances chemotherapy-induced apoptosis in high DDB2-expressing cancer cells. (A) Treating with lapatinib and chemotherapy enhanced the chemosensitivity of HuCCT1 cancer cells. Data was shown as the means ± SD. *p<0.05; **p<0.01; ***p<0.001 vs control group, Student's *t*-test. (B) The raw data of Figure 5E.

Fig. S9

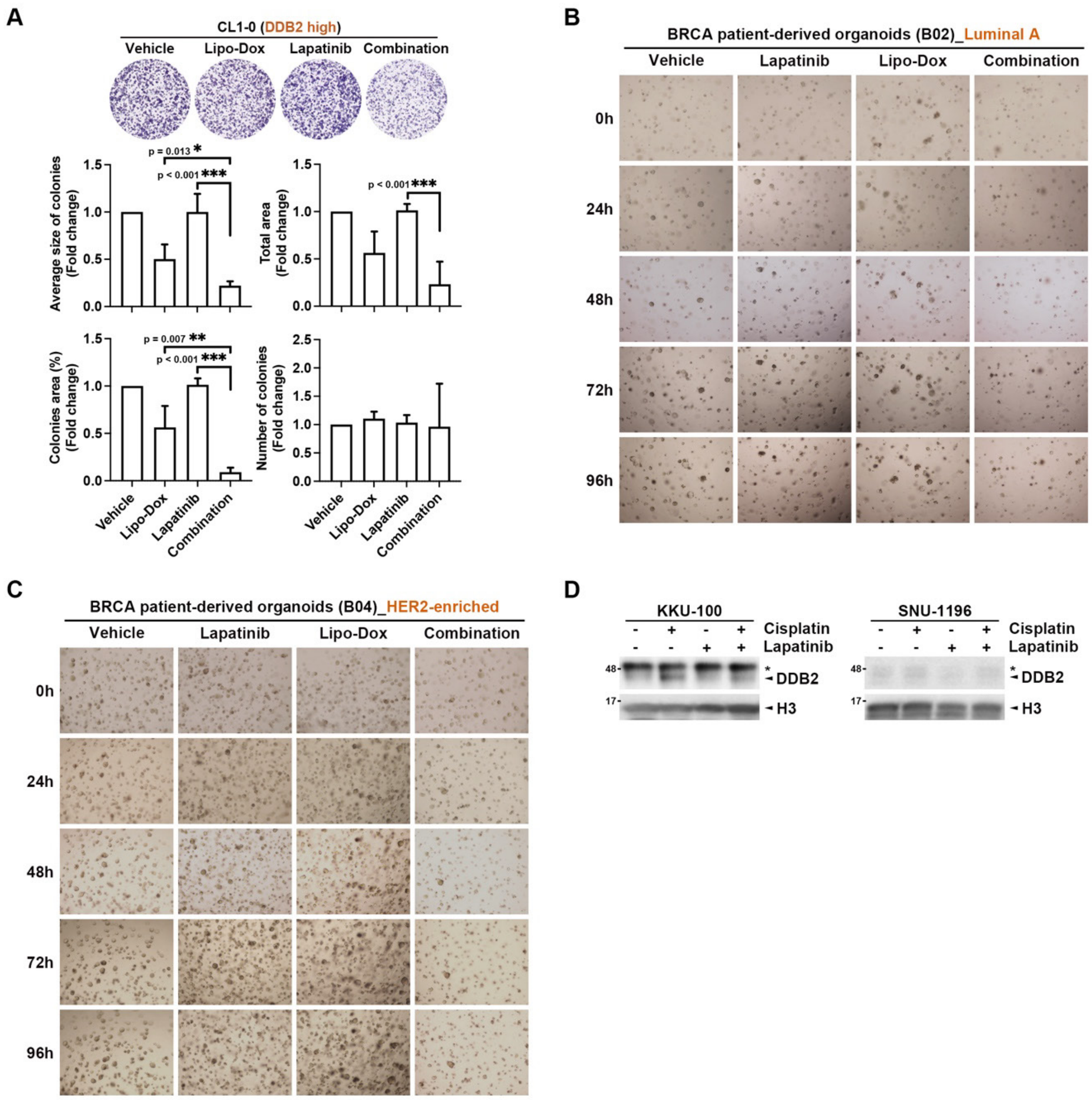

Figure S9. The anti-proliferative activity of Lipo-Dox is enhanced by lapatinib in patient-derived organoids. (A) The clonogenic assay was used to analyze the cell proliferation of CL1-0 cancer cells with indicated treatments with Lipo-Dox (0.2 μM) and/or lapatinib (1 μM) for 7 days. Quantitation data was calculated with ImageJ software. Data was shown as the means ± SD. *p<0.05; **p<0.01; ***p<0.001 vs control group, Student's *t*-test. (B-D) The raw data of Figure 6E. (E) Cholangiocarcinoma cells were treated with cisplatin (25 μM), either alone or in combination with lapatinib (1 μM) in a time-dependent manner. Total lysates were collected for western blot assay.
